# Supplementary material for: A systematic review and network meta-analysis of randomized controlled trials of well-being-focused interventions
Source: Nat Hum Behav. 2026 Jan 2;10(4):715–26. doi: 10.1038/s41562-025-02369-1 (PMC13120999; doi:10.1038/s41562-025-02369-1)
Supplement: Supplementary file 1 — All supplementary materials including reference list of included studies, additional tables and additional reporting of the sensitivity analyses. [file 41562_2025_2369_MOESM1_ESM.pdf]

nature human behaviour

Supplementary information

https://doi.org/10.1038/s41562-025-02369-1

A systematic review and network meta-analysis of randomized controlled trials of well-being-focused interventions

In the format provided by the authors and unedited

Table of Contents

1. **Eligibility and methods**
   1.1. Search Strategy
   1.2. Included studies characteristics
   1.3. Included studies references
   1.4. Cochrane risk of bias for individual studies using RoB 2
2. **Main Results**
   2.1. Original Intervention node labels
   2.2. Node Adaptations
      2.2.1. Initial assessment of transitivity prior to node exclusion (box plot distribution of potential effect modifier: age)
      2.2.2. Initial assessment of local inconsistency prior to node exclusion
      2.2.3. Rationale for node adaptions made based on assessments
   2.3. Characteristics of Included Studies Across the Different Comparisons
   2.4. Final assessment of local inconsistency following node exclusion
3. **Moderator Analyses using Meta-regressions**
   3.1. Table summary of results
   3.2. Intervention x Moderator interactions
4. **Sensitivity analyses**
   4.1. Sensitivity Analyses using Meta-Regression
   4.2. Sensitivity using alternative NMA models
5. **Confidence in Meta-Analysis Judgements (CINEMA)**
6. **Funnel Plots**

1. **Eligibility and Methods**

1.1. Search strategy

Medline (Via Ebsco)

1. TI (Well-being OR Wellbeing OR Positive affect OR Positive Emotion OR Wellness OR happy OR happiness or flourish* OR resilienc*) OR AB (Well-being OR Wellbeing OR Positive affect OR Positive Emotion OR Wellness OR happy OR happiness or flourish* OR resilienc*)
2. TI (life N1 satisfaction) OR AB (life N1 satisfaction)
3. #1 OR #2
4. TI (“Randomized controlled trial*” OR “Randomised controlled trial*” OR RCT OR “Clinical trial*” OR “Controlled clinical trial*”) OR AB (“Randomized controlled trial*” OR “Randomised controlled trial*” OR RCT OR “Clinical trial*” OR “Controlled clinical trial*”)
5. MH "Randomized Controlled Trials as Topic"
6. MH "Controlled Clinical Trials as Topic"
7. #4 OR #5 OR #6
8. TI (Mindfulness OR “Mindfulness based intervention*” OR “Mindfulness based stress reduction” OR MBSR OR MBI OR Compassion OR Meditation OR “Acceptance and Commitment Therapy” OR ACT) OR AB (Mindfulness OR “Mindfulness based intervention*” OR “Mindfulness based stress reduction” OR MBSR OR MBI OR Compassion OR Meditation OR “Acceptance and Commitment Therapy” OR ACT)
9. MH “Mindfulness+”
10. MH “Empathy”
11. MH “Meditation”
12. MH “Acceptance and Commitment Therapy”
13. TI (“Positive psychology” OR “Positive psychotherapy” OR PPI OR Strength* OR Gratitude OR Optimism OR Meaning OR Savouring OR Hope) OR AB (“Positive psychology” OR “Positive psychotherapy” OR PPI OR Strength* OR Gratitude OR Optimis* OR Meaning OR Savouring OR Hope)
14. MH “Psychology, Positive”
15. MH “Optimism”
16. MH “Hope”
17. TI (intervention* N1 (Nature OR outdoor*)) OR AB (intervention* N1 (Nature OR outdoor*))

18. TI (Ecotherap* OR Greenspace* OR “green space*” OR “blue space*” OR bluespace* OR “Green therap*” OR “natural environment” OR “Forest bathing” OR “forest therap*” OR “Shinrin Yoku” OR Horticultur* OR Garden* OR Conservation OR Allotment OR Beach* OR Countryside) OR AB (Ecotherap* OR Greenspace* OR “green space*” OR “blue space*” OR bluespace* OR “Green therap*” OR “natural environment” OR “Forest bathing” OR “forest therap*” OR “Shinrin Yoku” OR Horticultur* OR Garden* OR Conservation OR Allotment OR Beach* OR Countryside)
19. MH “Relaxation therapy”
20. MH “Parks, Recreational”
21. MH “Horticulture+”
22. TI (“Physical activit*” OR “Health promotion” OR “Health education” OR “Healthy Lifestyle” OR “Isometric training” OR “isometric program” OR “isometric conditioning” OR “isometric exercise*” OR “strength training” OR “strength conditioning” OR “strength program” OR “aerobic training” OR “aerobic exercise” OR “aerobic conditioning” OR “physical exercise” OR “stretching” OR “movement program” OR workout) OR AB (“Physical activit*” OR “Health promotion” OR “Health education” OR “Healthy Lifestyle” OR “Isometric training” OR “isometric program” OR “isometric conditioning” OR “isometric exercise*” OR “strength training” OR “strength conditioning” OR “strength program” OR “aerobic training” OR “aerobic exercise” OR “aerobic conditioning” OR “physical exercise” OR “stretching” OR “movement program” OR workout)
23. MH “Exercise”
24. MH “Health promotion”
25. MH “Health education”
26. MH “Healthy lifestyle”
27. TI ((Intervention OR Group* OR Club*) N1 (Support OR Social or self-help OR “self help”)) OR AB ((Group* OR Club*) N1 (Support OR Social OR “self-help”))
28. TI (“Social identity” OR “group identi*” OR “team identi*” OR “club identi*” OR “work identi*”) OR AB (“Social identity” OR “group identi*” OR “team identi*” OR “club identi*” OR “work identi*”)
29. MH “social identity+”
30. S8 OR S9 OR S10 OR S11 OR S12 OR S13 OR S14 OR S15 OR S16 OR S17 OR S18 OR S19 OR S20 OR S21 OR S22 OR S23 OR S24 OR S25 OR S26 OR S27 OR S28 OR S29
31. S3 AND S7 AND S30

Search results March 2023 = 3776

PsycINFO (Via Ebsco)

1. TI (Well-being OR Wellbeing OR Positive affect OR Positive Emotion OR Wellness OR happy OR happiness or flourish* OR resilienc*) OR AB (Well-being OR Wellbeing OR Positive affect OR Positive Emotion OR Wellness OR happy OR happiness or flourish* OR resilienc*)
2. TI (life N1 satisfaction) OR AB (life N1 satisfaction)
3. #1 OR #2
4. TI (“Randomized controlled trial*” OR “Randomised controlled trial*” OR RCT OR “Clinical trial*” OR “Controlled clinical trial*”) OR AB (“Randomized controlled trial*” OR “Randomised controlled trial*” OR RCT OR “Clinical trial*” OR “Controlled clinical trial*”)
5. TI (Mindfulness OR “Mindfulness based intervention*” OR “Mindfulness based stress reduction” OR MBSR OR MBI OR Compassion OR Meditation OR “Acceptance and Commitment Therapy” OR ACT) OR AB (Mindfulness OR “Mindfulness based intervention*” OR “Mindfulness based stress reduction” OR MBSR OR MBI OR Compassion OR Meditation OR “Acceptance and Commitment Therapy” OR ACT)
6. DE “Mindfulness”
7. DE “Empathy”
8. DE “Meditation”
9. DE “Acceptance and Commitment Therapy”
10. TI (“Positive psychology” OR “Positive psychotherapy” OR PPI OR Strength* OR Gratitude OR Optimism OR Meaning OR Savouring OR Hope) OR AB (“Positive psychology” OR “Positive psychotherapy” OR PPI OR Strength* OR Gratitude OR Optimis* OR Meaning OR Savouring OR Hope)
11. DE “Positive Psychology”
12. DE “Optimism”
13. DE “Hope”
14. TI (intervention* N1 (Nature OR outdoor*)) OR AB (intervention* N1 (Nature OR outdoor*))
15. TI (Ecotherap* OR Greenspace*OR “green space*” OR “blue space*” OR bluespace* OR “Green therap*” OR “natural environment” OR “Forest bathing” OR “forest therap*” OR “Shinrin Yoku” OR Horticultur* OR Garden* OR Conservation OR Allotment OR Beach* OR Countryside) OR AB (Ecotherap* OR Greenspace* OR “green space*” OR “blue space*” OR bluespace* OR “Green therap*” OR “natural environment” OR “Forest bathing” OR “forest therap*” OR “Shinrin Yoku” OR Horticultur* OR Garden* OR Conservation OR Allotment OR Beach* OR Countryside)
16. DE “Relaxation therapy”
17. DE “Horticulture Therapy”
18. TI (“Physical activit*” OR “Health promotion” OR “Health education” OR “Healthy Lifestyle” OR “Isometric training” OR “isometric program” OR “isometric conditioning” OR “isometric exercise*” OR “strength training” OR “strength conditioning”

OR “strength program” OR “aerobic training” OR “aerobic exercise” OR “aerobic conditioning” OR “physical exercise” OR “stretching” OR “movement program” OR workout) OR AB (“Physical activit*” OR “Health promotion” OR “Health education” OR “Healthy Lifestyle” OR “Isometric training” OR “isometric program” OR “isometric conditioning” OR “isometric exercise*” OR “strength training” OR “strength conditioning” OR “strength program” OR “aerobic training” OR “aerobic exercise” OR “aerobic conditioning” OR “physical exercise” OR “stretching” OR “movement program” OR workout)
19. DE “Exercise”
20. DE “Health promotion”
21. DE “Health education”
22. TI ((Intervention OR Group* OR Club*) N1 (Support OR Social or self-help OR “self help”)) OR AB ((Group* OR Club*) N1 (Support OR Social or self-help OR “self help”))
23. TI (“Social identity” OR “group identi*” OR “team identi*” OR “club identi*” OR “work identi*”) OR AB (“Social identity” OR “group identi*” OR “team identi*” OR “club identi*” OR “work identi*”)
24. DE “social identity”
25. S5 OR S6 OR S7 OR S8 OR S9 OR S10 OR S11 OR S12 OR S13 OR S14 OR S15 OR S16 OR S17 OR S18 OR S19 OR S20 OR S21 OR S22 OR S23 OR S24
26. S3 AND S4 AND S25

Results March 2023 = 1608

Scopus (Via Elsevier)

1. TITLE-ABS-KEY (Well-being OR Wellbeing OR Positive affect OR Positive Emotion OR Wellness OR happy OR happiness or flourish* OR resilienc*)
2. TITLE-ABS-KEY (life W/1 satisfaction)
3. #1 OR #2
4. TITLE-ABS-KEY (“Randomized controlled trial*” OR “Randomised controlled trial*” OR RCT OR “Clinical trial*” OR “Controlled clinical trial*”)
5. TITLE-ABS-KEY (Mindfulness OR “Mindfulness based intervention*” OR “Mindfulness based stress reduction” OR MBSR OR MBI OR Compassion OR Meditation OR “Acceptance and Commitment Therapy” OR ACT)
6. TITLE-ABS-KEY (“Positive psychology” OR “Positive psychotherapy” OR PPI OR Strength* OR Gratitude OR Optimism OR Meaning OR Savouring OR Hope)
7. TITLE-ABS-KEY (intervention* W/1 (Nature OR outdoor*))

8. TITLE-ABS-KEY (Ecotherap* OR Greenspace* OR “green space*” OR “blue space*” OR bluespace* OR “Green therap*” OR “natural environment” OR “Forest bathing” OR “forest therap*” OR “Shinrin Yoku” OR Horticultur* OR Garden* OR Conservation OR Allotment OR Beach* OR Countryside)
9. TITLE-ABS-KEY (“Physical activit*” OR “Health promotion” OR “Health education” OR “Healthy Lifestyle” OR “Isometric training” OR “isometric program” OR “isometric conditioning” OR “isometric exercise*” OR “strength training” OR “strength conditioning” OR “strength program” OR “aerobic training” OR “aerobic exercise” OR “aerobic conditioning” OR “physical exercise” OR “stretching” OR “movement program” OR workout)
10. TITLE-ABS-KEY ((Intervention OR Group* OR Club*) W/1 (Support OR Social or self-help OR “self help”))
11. TITLE-ABS-KEY (“Social identity” OR “group identi*” OR “team identi*” OR “club identi*” OR “work identi*”)
12. S5 OR S6 OR S7 OR S8 OR S9 OR S10 OR S11
13. S3 AND S4 AND S12

Results March 2023 = 2100

CENTRAL (Via Cochrane)

1. (Well-being OR Wellbeing OR Positive affect OR Positive Emotion OR Wellness OR happy OR happiness or flourish* OR resilienc*):ti,ab,kw
2. (life NEAR/1 satisfaction):ti,ab,kw
3. #1 OR #2
4. (“Randomized controlled trial*” OR “Randomised controlled trial*” OR RCT OR “Clinical trial*” OR “Controlled clinical trial*”):ti,ab,kw
5. MeSH descriptor [Randomized Controlled Trial] this term only
6. MeSH descriptor [Controlled Clinical Trial] this term only
7. #4 OR #5 OR #6
8. (Mindfulness OR “Mindfulness based intervention*” OR “Mindfulness based stress reduction” OR MBSR OR MBI OR Compassion OR Meditation OR “Acceptance and Commitment Therapy” OR ACT):ti,ab,kw
9. MeSH descriptor [Mindfulness] this term only
10. MeSH descriptor [Empathy] this term only
11. MeSH descriptor [Meditation] this term only
12. MeSH descriptor [Acceptance and Commitment Therapy] this term only

13. (“Positive psychology” OR “Positive psychotherapy” OR PPI OR Strength* OR Gratitude OR Optimism OR Meaning OR Savouring OR Hope):ti,ab,kw
14. MeSH descriptor [Psychology, Positive] this term only
15. MeSH descriptor [Optimism] this term only
16. MeSH descriptor [Hope] this term only
17. (intervention* NEAR/1 (Nature OR outdoor*)):ti,ab,kw
18. (Ecotherap* OR Greenspace* OR “green space*” OR “blue space*” OR bluespace* OR “Green therap*” OR “natural environment” OR “Forest bathing” OR “forest therap*” OR “Shinrin Yoku” OR Horticultur* OR Garden* OR Conservation OR Allotment OR Beach* OR Countryside):ti,ab,kw
19. MeSH descriptor [Relaxation Therapy] this term only
20. MeSH descriptor [Parks, Recreational] this term only
21. MeSH descriptor [Horticultural] explode all trees
22. (“Physical activit*” OR “Health promotion” OR “Health education” OR “Healthy Lifestyle” OR “Isometric training” OR “isometric program” OR “isometric conditioning” OR “isometric exercise*” OR “strength training” OR “strength conditioning” OR “strength program” OR “aerobic training” OR “aerobic exercise” OR “aerobic conditioning” OR “physical exercise” OR “stretching” OR “movement program” OR workout):ti,ab,kw
23. MeSH descriptor [Exercise] this term only
24. MeSH descriptor [Health Promotion] this term only
25. MeSH descriptor [Healthy Lifestyle] this term only
26. ((Intervention OR Group* OR Club*) NEAR/1 (Support OR Social or self-help OR “self help”)):ti,ab,kw
27. (“Social identity” OR “group identi*” OR “team identi*” OR “club identi*” OR “work identi*”):ti,ab,kw
28. MeSH descriptor [Social identity] this term only
29. #8 OR #9 OR #10 OR #11 OR #12 OR #13 OR #14 OR #15 OR #16 OR #17 OR #18 OR #19 OR #20 OR #21 OR #22 OR #23 OR #24 OR #25 OR #26 OR #27 OR #28
30. #3 AND #7 AND #29

Results March 2023 = 5757

1.2. Characteristics of included studies

Author	Country	Mean Age	Setting	Outcomes	Intervention 1	Intervention 2	Intervention 3	Intervention 4
Aeschbach, 2022	Germany	31.02	Workplace (Hospital)	FS, SCS	Theoretical information book on mindfulness	An 8-week mindfulness-based program		
Ahmad, 2020	Canada	24.8	University	16-item Quality of Life Scale (QOLS), FFMQ-SF	Wait list control	Mindfulness and CBT intervention		
Aikens, 2014	USA	Not reported	Workplace - (Corporation)	CD-RISC, FFMQ - Non Reactivity Scale	Wait list control	Mindfulness Programme		
Ajilchi, 2019	Iran	23.47	University	Mental Toughness Questionnaire (49 item)	No intervention	Mindfulness group		
Alexander, 2015	USA	46.38	Workplace (Hospital)	FMI	No intervention	Yoga		
Allexandre, 2016	USA	39.6	Workplace (Call Centre)	SF-36 Vitality	Wait list control	Mindfulness Program		
Amutio, 2015	Spain	47.31	Workplace (Healthcare)	FFMQ, SRSI3	Wait list control	MBSR mindfulness		
Ardi, 2021	Israel	30.91	Community and University	SWLS, CD-RISC	Wait list control	MBSR mindfulness		
Asuero, 2014	Spain	47.85	Workplace (Healthcare)	FFMQ, POMS Vigor	Wait list control	MBSR mindfulness		
Baker, 2008	Scotland	49.2	Community	PANAS-PA	No intervention	Walking program		

Basso, 2022	USA	30.72	Community	PANAS-PA	No intervention (maintain current exercise levels)	Exercise: Increase exercise regimen	
Bita, 2021	Iran	21.55	University	PWB, The Sports Mental Toughness Questionnaire (SMTQ)	No intervention - received usual sports coaching	Mindfulness Acceptance intervention	
Blasche, 2013	Austria	40.1	Workplaces (Various organisations)	Emotional well-being was assessed with a standardized German questionnaire (EBF)	Wait-List Control	Nordic walking	
Bohlmeijer, 2021	Netherla nds	48.6	Community	MHC-SF, GQ-6	Wait list control	Gratitude based writing exercises	Self acts of kindness
Bonde, 2022	Denmark	45.2	Workplace (School)	WHO-5, FFMQ	Wait list control	Mindfulness-based Stress Reduction (MSR)	
Borchardt, 2018	USA	18.94	University	PANAS-PA	Control - Sat quietly	Meditation Intervention	
Bowden, 2011	Not reported	Not reported	University	The Subjective Vitality Scale	Mindfulness classes	Iyengar Yoga Procedure	
Brinkmann, 2020	Germany	43.27	Workplace	FMI-SF, SCS	Wait list control	Mindfulness based intervention	
Brito-Pons, 2018	Chille	36.165	Community	SWLS, SHS	Wait list control	Compassion Training	
Cerna, 2020	Chile	30.9	Community	The Mental Health	Wait list control	MBSR mindfulness	

				Questionnaire (MHC)		
Champion, 2018	UK	39.13	Online	SWLS, The Wagnild Resilience Scale (WRS)	Wait list control	Headspace mindfulness introductory program
Chesak, 2015	USA	28.2	Workplace	CD-RISC	Educational lecture	Resilience Intervention
Christopher, 2018	USA	43.98	Workplace (Law enforcement)	FFMQ-SF, SCS-SF	No intervention control	Mindfulness training
Connolly, 2020	UK	40	Community	WEMWBS	No intervention	12 week exercise training DVD
Crain, 2017	Canada and USA	46.9	Workplace (School)	FFMQ	Wait list control	Mindfulness Training Program
Cruz-Ferreira, 2011	Portugal	40.7	University	SWLS	Wait list control	Pilates
Cruz-Ferreira, 2015	Portugal	71.95	Health centre	SWLS	No intervention control	Creative Dance Exercise program
Danitz, 2014	United States	21.13	University	Philadelphia Mindfulness Scale	Wait list control	Acceptance based Workshop
deVibe, 2018	Norway	23.8	University	FFMQ	Control - studies as usual	Mindfulness
Devillers-Réolon, 2022	France	22.13	University	WEMWBS	No intervention control	Mindfulness: daily online mindfulness
Díaz-Benito, 2022	Spain	34.31	Workplace	SF-36 Vitality	No intervention control	Exercise: aerobic and strength training

Duan, 2019	China	18.22	University	Brief Inventory of Thriving (BIT)	No intervention control	Character strengths intervention		
Dvořáková, 2017	USA	18.2	University	SWLS, SCS	No intervention control	Mindfulness program students		
Economides, 2018	Not reported	No mean reported	Online	SPANE	Audio mindfulness content	Headspace mindfulness app		
Edney, 2020	Australia	41	Online	PP	Wait list control	Increase daily step count		
Edwards, 2019	USA	21.43	University	Exercise Induced Feelings Inventory: positive engagement	Control - sit in lab	Walking	Meditation Group	Meditatio n then Walk
Engel, 2019	Germany	36.2	University (Laboratory)	WHOQOL-BREF	Control - maintained their standard exercise training	Functional high-intensity training		
EnriqueRoig, 2020	Ireland	26	University	Pemberton Happiness Index (PHI), CD-RISC	Wait list control	Space for resilience intervention		
Erogul, 2014	USA	23.5	University	SCS, The Resilience Scale (RS)	No intervention control	MBSR mindfulness		
Fernández-Portero, 2021	Spain	52.08	Community	SWLS, CD-RISC	Physical activity training	Mindfulness and acceptance-based intervention		

Fisher, 2004	USA	73.99	Community	SWLS	Education booklet control	Walking (outdoor)
Flook, 2013	USA	43.06	Workplace (School)	FFMQ-Non Reactivity Sub-Scale, SCS - Shared Humanity	Wait list control	MBSR Mindfulness
Galante, 2016	UK/USA	Not reported	Online	WEMWBS	Light Exercise (Video based)	Loving kindness meditation
Gerodimos, 2022	Greece	44.44	Workplace - hospital	Subjective Vitality Scale	No intervention	Chair based exercise group
Goldberg, 2020	United States	41.74	Online based	FFMQ	Waitlist Control	Awareness and Insight
Green, 2006	Austrailia	42.68	Community	SWLS	Waitlist Control	Life coaching group programme
Green, 2022	Pakistan	23.26	University	PP	Waitlist Control	Character strengths intervention
Gregoire, 2017	Canada	31.72	University	Well-Being Manifestations Measure Scale (WBMMS), FFMQ-SF	Waitlist Control	Four workshops based on ACT
Hajatnia, 2021	Iran	Not reported - all over 65	Elderly home	CD-RISC	No intervention control	Acceptance and commitment therapy
Harris, 2016	USA	43	Workplace (School)	FFMQ-Non Reactivity Sub-Scale, PANAS-PA	Wait list control	Community Approach to Learning Mindfully (CALM) program for educators

Heintzelman, 2019	USA	45.36	University	SWLS, SPANE	Waitlist Control	Enduring Happiness and Continued Self-Enhancement (ENHANCE)	
Hendriks, 2020	Suriname, Caribbean	36.3	Workplace	MHC-SF, PANAS-PA	Waitlist Control	Multi-component positive psychology intervention	
Hilcove, 2021	USA	42.45	Workplace (Healthcare)	SF-36 Vitality	No intervention control	Group yoga sessions	
Hirshberg, 2018	USA	19.29	University	PANAS-PA	Gratitude practice	Breath awareness	Loving-kindness meditation
Hirshberg, 2022	USA	42.5	Online (App)	WHO-5, SCS-SF	Waitlist Control	Healthy Minds Program	
Ho, 2022	Hong Kong, China	34.76	University	SWLS, Chinese Affect Scale (CAS-PA)	Waitlist Control	Nature Contact	
Hollingsworth, 2022	USA	44.6	Online	GQ-6, Adult Hope Scale	Inactive Control	Tiny habits for gratitude	
Hu, 2017	China	53.38	Community	SWLS	Waitlist Control	Exercise intervention: health education and walking	
Huang, 2021	China	20.55	University/College	SCS	Waitlist Control	Self-compassion Intervention	
Hunsinger, 2018	USA	43.98	Workplace (Law enforcement)	CD-RISC, SCS-SF	No intervention control	Mindfulness-based resilience training (MBRT)	
Hunt, 2018	USA	19.3	University	PANAS-PA	No intervention control	Mindfulness Training	Yoga

Hwang, 2019	Austrailia	42.34	Workplace (School)	FFMQ-SF, SCS-SF	Waitlist Control	Reconnected - A mindfulness-based training program
Ivtzan, 2016	UK, USA, Canada, Austrailia	40.82	Online	The Pemberton Happiness Index (PHI), GQ6	Waitlist Control	The Positive Mindfulness Program
Ivtzan, 2018	Hong Kong and UK	31.5	Online	FMI	Waitlist Control	The Mindfulness Based Flourishing program
Janssen, 2022	The Netherla nds	49	Workplace (School)	FFMQ	Waitlist Control	Mindfulness-Based Stress Reduction (MBSR)
Janzarik, 2022	Germany	46.95	Workplace (Healthcare)	SWLS, WHO-5	Waitlist Control	Psychodynamic and CBT based therapy group
Jarukasemthawee, 2019	Thailand	20.5	University	The Eudemonic Well-Being Scale, FMI	Waitlist Control	Insight-Based Mindfulness Program
Jazaieri, 2013	USA	43.33	Community	SCS	Waitlist Control	Compassion Cultivation Training
Jazaieri, 2014	USA	43.08	Community	SHS, Kentucky Inventory of Mindfulness Scale (KIMS)	Waitlist Control	Compassion cultivation training (CCT)
Jennings, 2019	USA	Median age reported: 40	Workplace (School)	FFMQ	Waitlist Control	CARE program
Jones, 2019	USA	18.97	University	FFMQ, Coping Flexibility Scale	Waitlist Control	Mindfulness Meditation Intervention

Josefsson, 2014	Sweden		Workplace	PWB-SF, FFMQ	No intervention	Mindfulness Meditation: Standard sitting
Juul, 2021	Denmark	26.35	University	WHO-5, BRS	Waitlist Control	Mindfulness-Based Stress Reduction (MBSR)
Kadian, 2022	India	19.31	University/College	Brief Resilient Coping Scale	Resilience self-help booklet group	Brief resilience intervention program
Kaemmerer, 2022	France	39.24	University	SCS	Waitlist Control	Mindfulness
Kam, 2022	USA	29.9	Online	PANAS-PA	Waitlist Control	Mindfulness Training - Headspace
Kamegaya, 2014	Japan	74.9	Community centre	The Satisfaction in Daily Life	Waitlist Control	Group Physical Activity Programme
Kang, 2022	China	36.96	Online	WHO-5, PANAS-PS	Waitlist Control	Short-term audio-based mindfulness
Karing, 2021	Germany	22.68	University	FFMQ	Waitlist Control	Mindfulness training
Kariyawasam, 2022	UK & Sri Lanka	Not reported	Online	WEMWBS	Wait list control	Compassionate Mind Training
Khazaee-Pool, 2015	Iran	71	Community	OHI	No intervention	Exercise intervention
Kimura, 2010	Japan	74	University	SF-36-Mental Health, SF-36 Vitality	Health education group control	Exercise intervention
Kloos, 2022	Netherlands	53.2	Online (App)	MHC-SF	Wait-list control	Gratitude Intervention App
Ko, 2018	USA	19.78	University	SCS	Wait-list control	Compassion Seminar
Kosugi, 2021	Japan	46.8	University	SWLS, FS	Wait-list control	MBCT

Krekel, 2021	UK	Not reported	Community	Santa Clara Brief Compassion Scale	Wait-list control	Exploring what matters course
Kuhlthau, 2020	USA	45	communtiy	Current Experience Scale (CES), PANAS-PA	Wait-list control	Mind body intervention
Kwon, 2015	South Korea	71.35	Community	SF-8 TM - Mental Component Scale	No intervention	Wheel of Wellness counseling
LeBlanc, 2017	UK	36.68	University	SWLS	No intervention	Workshops focussed on emotion regulation, expressive writing, mindfulness, and self-talk and cue-controlled progressive muscle relaxation.
Lee, 2016	UK	33	Community	Adapted General Well-Being Index (AGWBI)	The control group received	Education and Walking Group
Lee, 2019	Taiwan	61	Community	CHI 20-item version	Walking Exercise	Walking exercise + positive psych coaching
Lee, 2021	China	71.55	Community	SWEMWBS, SCS	Waitlist Control	Mindfulness-based Stress Reduction
Li, 2001	USA	73.2	Community	SWLS, PANAS-PA	Waitlist Control	Exercise intervention : Tai Chi
Li, 2015	China	20.78	University	WHOQOL-BREF	Control - usual	Baduanjin Exercise -

					physical activity	
Liu, 2020	China	29.26	Workplace (Airline)	SWLS, State Mindfulness Scale (SMS)	No intervention control	Loving kindness meditation
Liu, 2022 A	China	38.83	Workplace	PANAS-PA	Waitlist Control	Loving-Kindness Meditation
Liu, 2022 b	China	25.26	University	SWLS, SPANE	Health education	Moderate physical activity
Loewenthal, 2021	USA	29.23	Workplace (Hospital)	FFMQ, RS-14	Wait list control	Yoga
Lorenz, 2022	Germany	23.95	University	BRS, PANAS-PA	Wait list control	Self guided positive psychology
Ly, 2017	Sweden	23.25	Online	SWLS, FS	Wait list control	Chatbot Intervention: Positive Psychology
Maatouk, 2018	Germany	52.11	Workplace (Hospital)	WHOQOL-BREF	Wait list control	Intervention: Successful Ageing
Mackenzie, 2006	Canada	46.7	Workplace (Hospital)	SWLS	Wait list control	Shortened Version of MBSR
Mak, 2015	China	22.8	University	WHO-5, SWLS	Waitlist Control	Health Action Process Approach
Manotas, 2014	Colombia	39.05	Workplace (Hospital)	FFMQ	No intervention	Mindfulness-based Stress Reduction (MBSR)
Mascaro, 2021	USA	Not reported	Workplace	CD-RISC	Waitlist Control	Compassion-Centered Spiritual Health
Matos, 2022	Portugal	51.35	Workplace (School)	Types of Positive Affect Scale - Safe PA, Compassionate Engagement	Waitlist Control	The Compassionate Mind Training

				and Action Scales		
Matvienko-Sikar, 2017	Ireland	33.87	Communtiy	SWLS	No intervention	Gratitude & Mindfulness Intervention
Maurer, 2020	Germany	24.19	University	PANAS-PA	No intervention - usual lifestyle	Endurance Exercise Intervention
McConachie, 2014	UK	Not reported	Workplace	WEMWBS	Waitlist Control	Acceptance and mindfulness workshop
McGonagle, 2020	USA	42.62	Workplace (Healthcare)	24-item Psychological Capital Questionnaire	Waitlist Control	Positive psychology coaching
Miller, 2015	UK	36.46	University	SWLS, OHQ	Wellbeing articles	Gratitude
Mirabito, 2022	USA	21.39	Online	PWB, PANAS	Waitlist Control	Mindfulness Intervention
Montaner, 2022	Spain	41.1	Workplace (Hospital)	SWLS	Waitlist Control	Acceptance and Commitment Therapy
Morledge, 2013	USA	Not reported	Online	PWB-Self Acceptance Sub-Scale, Subjective Vitality Scale	Waitlist Control	Internet-Based Stress Management Program
Mountain, 2017	UK	72.1	Community	SF-36 Mental Health, BRS	No intervention	Lifestyle Matters Intervention
Muller, 2016	Germany	39.9	Workplace (Hospital)	WHO-5	Waitlist Control	Intervention: Selection, optimisation, and compensation (SOC)

Murray, 2021	USA	40.62	Community	WHO-5	Waitlist Control	Education and physical activity	
Myers, 2017	USA	41.76	Workplace (University)	I COPE	Control - wellbeing info website access	Online wellness intervention	
Nadler, 2020	USA	Not reported	Workplace	FFMQ, PANAS-PA	Waitlist Control	Online Workplace-Based Mindfulness Training:	
Nedeljkovic, 2012	Switzerla nd	35.48	Unreported	Self-Compassion Scale (SCS)	Waitlist Control	Taiji classes	
Neff, 2013	USA	50	Communtiy	SCS, SWLS	Waitlist Control	Mindful Self compassion	
Neumeier, 2017	Austrailia	41.16	Community	SHS & PANAS	Waitlist Control	Self administered PERMA program	Gratitude
Ng, 2016	Singapor e	67.01	Community	PWB	Waitlist Control	Horticultural Therapy	
Nguyen, 2018	Taiwan	22	University	The 4 items assessing user happiness were adapted from Lyubomirsky and Lepper (1999)	No intervention control	Exergames	
Nielsen, 2021	Canada	46	Workplace	BRS, PANAS-PA	Waitlist Control	The Anxious Lawyer: Book	
Noradechanunt, 2017	Australia	66.66	Communities	SF-36 Mental Health, Vitality	Control Group - received	Thai Yoga	
Norman, 2010	Australia	29.7	Hospital	PABS	Control: Physical education only	Intervention: Exercise and Education	

O'Leary, 2015	Ireland	28.35	Home/Online	4-item Subjective Happiness Scale	Waitlist Control	Gratitude Intervention	Mindfulness Intervention
Odou, 2013	Australia	34	Online	WEMWBS, PANAS-PA	No intervention control	Three Good Things	
Oken, 2006	USA	72.1	University	SF-36 Mental Composite	Waitlist Control	Hatha Yoga Class	Walking Exercise
Oliver, 2018	United Kingdom	Not reported	Workplace	SWLS, FS	Wait-List Control	Online Goal-Setting and Planning	
Pacanowski, 2020	USA	19.63	University/College	PANAS-PA, FFMQ	Control Group	Yoga Group	
Page, 2013	Australia	39.7	Workplace	SWLS & PANAS	Inactive control group	Working for Wellness Program	
Pan, 2019	Taiwan	32.8	Hospital	FFMQ	Education only control	Mindfulness-based childbirth and parenting	
Pang, 2019	Switzerland	44.2	Workplace	WHO-5	Waitlist Control	Mindfulness-based strengths	Mindfulness-based stress reduction
Passmore, 2022	Canada	46.75	Community	SWLS, PANAS-PA	Waitlist Control	Nature intervention	
Payne, 2020	Australia	31.2	University	SWLS	Waitlist Control	Nature Intervention group	
Perez-Blasco, 2016	Spain	63.56	Community	BRCS	No intervention control	Mindfulness	
Proyer, 2016	Switzerland	45.66	University	AHI, Orientations to Happiness Questionnaire	Waitlist Control	Positive Psychology	

Rababah, 2022	Jordan	20.1	University	FFMQ	Wait list control	Modified version of motivational interviewing	
Read, 2016	Australia	52.8	Community	WEMWBS	Waitlist Control	Single-session Behavioural Activation	
Recabarren, 2019	Switzerland	21.34	University	WHOQOL-BREF, SCS-SF	Control	Stress Prevention Intervention	
Rich, 2021	UK	Not reported	University	FFMQ-SF	Waitlist Control	Headspace mindfulness-based meditation training	
Rodríguez-Jiménez, 2022	Spain	43.3	Workplace (University)	SWLS, WHO-5	Waitlist Control	Hatha Yoga	
Roeser, 2013	Canada and USA	46.9	Workplace (School)	FFMQ	Waitlist Control	Mindfulness Training Program	
Santos, 2022	Portugal	44.43	Workplace (Care Home)	SCS, CS	Control	Compassionate Mind Training program	
Schulte-Frankenfeld, 2022	The Netherlands	24.75	University	Questionnaire for the Assessment of Happiness, FMI-14	Waitlist Control	Balloon Meditation App	
Seear, 2013	Australia	33.15	Online	WEMWBS, PANAS-PA	No-activity control group	Three good things	
Senf, 2013	Malaysia	20.3	University	Steen Happiness Index	Control Group	Strength-based intervention	
Seppälä, 2020	USA	19.67	University	PWB - Purpose Scale, SWLS	Inactive Control	SKY Campus Happiness	Mindfulness-Based Stress Reduction

Shapiro, 2011	USA	18.73	University	SWLS & PANAS, SCS	Waitlist Control	Mindfulness-based Stress Reduction (MBSR):
Sommers-Spijkerman, 2018	The Netherlands	52.87	Online	MHC-SF, PANAS-PA	Waitlist Control	Compassion-Focused Therapy
Sood, 2014	USA	47.75	Workplace	CD-RISC	Waitlist Control	Stress Management and Resiliency
Spence, 2007	Australia	38.59	Community	SWLS, PWB-Purpose	Waitlist control group	Professional Coaching
Spence, 2019	Australia	42.47	University	PWB, SWLS	No intervention	Mindfulness meditation
Steinhardt, 2008	USA	22.7	University	CD-RISC, PANAS-PA	Waitlist Control	Resilience intervention
Strauss, 2021	UK	43.93	Workplace (Healthcare)	SWEMWBS, FFMQ-SF	Waitlist Control Group	Mindfulness + CBT Intervention
Sturm, 2022	USA	Median age: 75	Community	SWLS	Active Control - Walking	Awe Walk
Taylor, 2014	UK	28.61	University	SWLS, FFMQ	wait list control	Mindfulness book self help
Taylor, 2022	UK	40.53	Workplace (Healthcare)	SWEMWBS, SCS-SF	Psychoeducation platform	Headspace
Terblanche, 2022	UK	22	University (Business School)	WEMWBS, BRS	Control group	AI coach (Vici)
Timlin, 2017	Northern Ireland, UK	28.19	Community Centre	PANAS-PA	Waitlist Control	Dru Yoga Programme
Trombka, 2021	Brazil	42.26	Workplace (Law enforcement)	WHOQOL-BREF	Waitlist Control	Mindfulness-based health promotion (MBHP)

Tsang, 2021	China	39.54	Workplace (School)	SWLS, PANAS-PA	Waitlist Control	A school-based mindfulness training	
Van Roie, 2017	Belgium	82.12	Community	Marcoen Scale	No intervention	Structured ergometer-cycling training program	
vanDijk, 2017	The Netherla nds	23.5	University	MHC-SF, Life Satisfaction Questionnaire (LiSat-9)	No intervention	Mindfulness-Based Stress Reduction	
vanEmmerik, 2018	The Netherla nds	44.7	Online (App)	WHOQOL-BREF, FFMQ	Waitlist Control	The VGZ Mindfulness Coach	
Verweij, 2018	The Netherla nds	31.2	University	SCS-SF, MHC-SF	Waitlist control	MBSR Intervention	
Viskovich, 2020	Australia	26.85	University	MHC-SF, SWLS	Waitlist Control	Web based ACT intervention	
Wadhen, 2021	UK	42.45	Workplace	WEMWBS, Coping Self-Efficacy Scale (CSES-26)	Waitlist Control	Yoga	
Waelde, 2017	USA	59.6	Workplace	SWLS	Psychoeduca tion and telephone support	Inner Resources for Stress	
Wang, 2020	Hungary	24.96	University	SWLS	Control - maintain a sedentary lifestyle	Walking intervention	
Welford, 2022	Sweden	72.5	Community	SWLS	Waitlist Control	Yoga	Aerobic Exercise - participant s

Weytens, 2014	Belgium	22.29	University	SWLS, SHS	Waitlist Control	Positive Emotion Regulation (PER)	Loving-Kindness Meditation Program
Wilke, 2022	Argentina, Austria, Brazil, Chile, Germany, Italy, Ireland, South Africa and Spain	32.8	Home	WHO-5	Inactive control group	Tele-exercise: moderate intensity livestreamed	
Wingert, 2022	USA	18.95	University	PP	Waitlist Control	Mindfulness-based strengths practice	
Xiong, 2022	USA	25.58	University	PP, FFMQ	Waitlist Control Group	Mindfulness-based well-being group	
Xu, 2021	Australia	Not reported	Workplace (Hospital)	WEMWBS	Waitlist Control	Headspace App	
Yang, 2018	USA	25.11	University	General Well-Being Schedule (GWBS), FFMQ	Waitlist Control	Mindfulness Mobile Phone Application	
Yıldırım, 2022	Turkey	28.33	University Hospital	PWB	Relaxation control group	Mindfulness-based breathing and music	
Zeng, 2019	China	27.55	University	SWLS, Appreciative Joy Scale (AJS)	Waitlist Control	Appreciative Joy Meditation Intervention	

Zhang, 2019	China	22.5	University	FMI, PANAS-PA	Waitlist Control	Mindfulness Training and Homework
Zheng, 2022	China	27.29	University and Community	SWLS	Waitlist Control	Mindfulness-based positive Intervention
Zilcha-Mano, 2016	Israel	28.71	Online	SWLS, PANAS-PA	No intervention	Mindfulness Intervention

1.3. References of included studies

Aeschbach, V.M., Fendel, J.C., Göritz, A.S., Schulze-Marmeling, C., & Schmidt, S. (2022). The effects of a tailored mindfulness-based program on the positive mental health of resident physicians—A randomized controlled trial. *Mindfulness*, *13*(5), 1292-1306. https://doi.org/10.1007/s12671-022-01876-w

Ahmad, F., El Morr, C., Ritvo, P., Othman, N., & Moineddin, R. (2020). An Eight-Week, Web-Based Mindfulness Virtual Community Intervention for Students' Mental Health: Randomized Controlled Trial. *JMIR mental health*, *7*(2), 15520. https://doi.org/10.2196/15520

Aikens, K.A., Astin, J., Pelletier, K.R., Levanovich, K., Baase, C.M., Park, Y.Y., & Bodnar, C.M. (2014) Mindfulness goes to work: Impact of an online workplace intervention. *Journal of Occupational and Environmental Medicine*, *56*, 721-731. https://doi.org/10.1097/JOM.0000000000000209

Ajilchi, B., Amini, H.R., Ardakani, Z.P., Zadeh, M.M., & Kisely, S. (2019). Applying mindfulness training to enhance the mental toughness and emotional intelligence of amateur basketball players. *Australasian psychiatry : bulletin of Royal Australian and New Zealand College of Psychiatrists*, *27*(3), 291-296. https://doi.org/10.1177/1039856219828119

Alexander, G.K., Rollins, K., Walker, D., Wong, L., & Pennings, J. (2015). Yoga for Self-Care and Burnout Prevention Among Nurses. *Workplace health & safety*, *63*(10), 462-70. https://doi.org/10.1177/2165079915596102

Allexandre, D., Bernstein, A.M., Walker, E., Hunter, J., Roizen, M.F., & Morledge, T.J. (2016). A Web-Based Mindfulness Stress Management Program in a Corporate Call Center: A Randomized Clinical Trial to Evaluate the Added Benefit of Onsite Group Support. *Journal of occupational and environmental medicine*, *58*(3), 254-264. https://doi.org/10.1097/JOM.0000000000000680

Amutio, A., Martínez-Taboada, C., Hermosilla, D., & Delgado, L.C. (2015). Enhancing relaxation states and positive emotions in physicians through a mindfulness training program: A one-year study. *Psychology, health & medicine*, *20*(6), 720-731. https://doi.org/10.1080/13548506.2014.986143

Ardi, Z., Golland, Y., Shafir, R., Sheppes, G., & Levit-Binnun, N. (2021) The Effects of Mindfulness-Based Stress Reduction on the Association Between Autonomic Interoceptive Signals and Emotion Regulation Selection. *Psychosom Med*, *83*(8), 852-862. https://doi.org/10.1097/PSY.0000000000000994

Asuero, A.M., Queraltó, J.M., Pujol-Ribera, E., Berenguera, A., Rodriguez-Blanco, T., & Epstein, R.M. (2014). Effectiveness of a mindfulness education program in primary health care professionals: a pragmatic controlled trial. *The Journal of continuing education in the health professions*, *34*(1), 4-12. https://doi.org/10.1002/chp.21211

Baker, G., Gray, S.R., Wright, A., Fitzsimons, C., Nimmo, M., Lowry, R., & Mutrie, N. (2008). The effect of a pedometer-based community walking intervention "Walking for Wellbeing in the West" on physical activity levels and health outcomes: a 12-week randomized controlled trial. *The international journal of behavioral nutrition and physical activity*, *5*, 44. https://doi.org/10.1186/1479-5868-5-44

Basso, J., Oberlin, D., Satyal, M., O'Brien, C., Crosta, C., Psaras, Z., Metpally, A., & Suzuki, W. (2022). Examining the Effect of Increased Aerobic Exercise in Moderately Fit Adults on Psychological State and Cognitive Function. https://doi.org/ 10.3389/fnhum.2022.833149

Bita, A., Mahmoud, M., Sahar, Z., & Steve, K. (2021). Effect of a mindfulness programme training on mental toughness and psychological well-being of female athletes. https://doi.org/ 10.1177/10398562211057075

Blasche, G., Pfeffer, M., Thaler, H., & Gollner, E. (2013). Work-site health promotion of frequent computer users: comparing selected interventions. https://doi.org/ 10.3233/WOR-121520

Bohlmeijer, E.T., Kraiss, J.T., Watkins, P., & Schotanus-Dijkstra, M. (2021). Promoting gratitude as a resource for sustainable mental health: Results of a 3-armed randomized controlled trial up to 6 months follow-up. *Journal of Happiness Studies: An Interdisciplinary Forum on Subjective Well-Being*, *22*(3), 1011-1032. https://doi.org/10.1007/s10902-020-00261-5

Bonde, E.H., Fjorback, L.O., Frydenberg, M., & Juul, L. (2022). The effectiveness of mindfulness-based stress reduction for school teachers: a cluster-randomized controlled trial. *European journal of public health*, *32*(2), 246-253. https://doi.org/10.1093/eurpub/ckab223

Borchardt, A., & Zoccola, P. (2018). Recovery from stress: An experimental examination of focused attention meditation in novices. *J. Behav. Med.*, *41*(6), 836-849. https://doi.org/10.1007/s10865-018-9932-9

Bowden, D., Gaudry, C., & Gruzelier, J. (2011). Acomparative randomised controlled trial of the effects of Brain Wave Vibration yoga, Iyengar yoga and Mindfulness training on mood and well-being. https://doi.org/ 10.1016/j.neulet.2011.05.135

Brinkmann, A.E., Press, S.A., Helmert, E., Hautzinger, M., Khazan, I., & Vagedes, J. (2020). Comparing Effectiveness of HRV-Biofeedback and Mindfulness for Workplace Stress Reduction: A Randomized Controlled Trial. *Applied psychophysiology and biofeedback*, *45*(4), 307-322. https://doi.org/10.1007/s10484-020-09477-w

Brito-Pons, G., Campos, D., & Cebolla, A. (2018). Implicit or explicit compassion? Effects of compassion cultivation training and comparison with mindfulness-based stress reduction. *Mindfulness*, *9*(5), 1494-1508. https://doi.org/10.1007/s12671-018-0898-z

Cerna, C., García, F.E., & Téllez, A. (2020). Brief mindfulness, mental health, and cognitive processes: A randomized controlled trial. *PsyCh journal*, *9*(3), 359-369. https://doi.org/10.1002/pchj.325

Champion, L., Economides, M., & Chandler, C. (2018). The efficacy of a brief app-based mindfulness intervention on psychosocial outcomes in healthy adults: A pilot randomised controlled trial. *PloS one*, *13*(12), e0209482. https://doi.org/10.1371/journal.pone.0209482

Chesak, S., Bhagra, A., Schroeder, D., Foy, D., Cutshall, S., & Sood, A. (2015). Enhancing resilience among new nurses: feasibility and efficacy of a pilot intervention.

Christopher, M.S., Hunsinger, M., Goerling, L.R.J., Bowen, S., Rogers, B.S., Gross, C.R., Dapolonia, E., & Pruessner, J.C. (2018) Mindfulness-based resilience training to reduce health risk, stress reactivity, and aggression among law enforcement officers: A feasibility and preliminary efficacy trial. *Psychiatry Research*, *264*, 104-115. https://doi.org/https://doi.org/10.1016/j.psychres.2018.03.059

Connolly, L.J., Scott, S., Morencos, C.M., Fulford, J., Jones, A.M., Knapp, K., Krustrup, P., Bailey, S.J., & Bowtell, J.L. (2020) Impact of a novel home-based exercise intervention on health indicators in inactive premenopausal women: a 12-week randomised controlled trial. *European journal of applied physiology*, *120*(4), 771-782. https://doi.org/10.1007/s00421-020-04315-7

Crain, T.L., Schonert-Reichl, K.A., & Roeser, R.W. (2017) Cultivating teacher mindfulness: Effects of a randomized controlled trial on work, home, and sleep outcomes. *Journal of occupational health psychology*, *22*(2), 138-152. https://doi.org/10.1037/ocp0000043

Cruz-Ferreira, A., Fernandes, J., Gomes, D., Bernardo, L.M., Kirkcaldy, B.D., Barbosa, T.M., & Silva, A. (2011) Effects of Pilates-based exercise on life satisfaction, physical self-concept and health status in adult women. *Women & health*, *51*(3), 240-255. https://doi.org/10.1080/03630242.2011.563417

Cruz-Ferreira, A., Marmeleira, J., Formigo, A., Gomes, D., & Fernandes, J. (2015) Creative Dance Improves Physical Fitness and Life Satisfaction in Older Women. *Res. Aging*, *37*(8), 837-855. https://doi.org/10.1177/0164027514568103

Danitz, S., & Orsillo, S. (2014) The Mindful Way Through the Semester: An Investigation of the Effectiveness of an Acceptance-Based Behavioral Therapy Program on Psychological Wellness in First-Year Students. *Behav. Modif.*, *38*(4), 549-566. https://doi.org/10.1177/0145445513520218

de Vibe, M., Solhaug, I., Rosenvinge, J.H., Tyssen, R., Hanley, A., & Garland, E. (2018) Six-year positive effects of a mindfulness-based intervention on mindfulness, coping and well-being in medical and psychology students; Results from a randomized controlled trial. *PloS one*, *13*(4), e0196053. https://doi.org/10.1371/journal.pone.0196053

Devillers-Réolon, L., Mascret, N., & Sleimen-Malkoun, R. (2022)Online Mindfulness Intervention, Mental Health and Attentional Abilities: A Randomized Controlled Trial in University Students During COVID-19 Lockdown. *Frontiers in psychology*, *13*, 889807. https://doi.org/10.3389/fpsyg.2022.889807

Díaz-Benito, V., Barriopedro Moro, M., Clemente Remón, Á., Santacruz Lozano, J., Hervás Pérez, J., & Vanderhaegen, F. (2022) Effects of worksite exercise intervention (PRODET®) on well-being at work and capability in performing work-related sedentary tasks: a pilot study. https://doi.org/ 10.3233/WOR-205340

Duan, W., Bu, H., Zhao, J., & Guo, X. (2019) Examining the Mediating Roles of Strengths Knowledge and Strengths Use in a 1-Year Single-Session Character Strength-Based Cognitive Intervention. *J. Happiness Stud.*, *20*(6), 1673-1688. https://doi.org/10.1007/s10902-018-0014-z

Dvořáková, K., Kishida, M., Li, J., Elavsky, S., Broderick, P.C., Agrusti, M.R., & Greenberg, M.T. (2017) Promoting healthy transition to college through mindfulness training with first-year college students: Pilot randomized controlled trial. *Journal of American college health : J of ACH*, *65*(4), 259-267. https://doi.org/10.1080/07448481.2017.1278605

Economides, M., Martman, J., Bell, M.J., & Sanderson, B. (2018) Improvements in Stress, Affect, and Irritability Following Brief Use of a Mindfulness-based Smartphone App: A Randomized Controlled Trial. *Mindfulness*, *9*(5), 1584-1593. https://doi.org/10.1007/s12671-018-0905-4

Edney, S.M., Olds, T.S., Ryan, J.C., Vandelanotte, C., Plotnikoff, R.C., Curtis, R.G., & Maher, C.A. (2020) A Social Networking and Gamified App to Increase Physical Activity: Cluster RCT. *American journal of preventive medicine*, *58*(2), e51-e62. https://doi.org/10.1016/j.amepre.2019.09.009

Edwards, M., & Loprinzi, P. (2019) Affective Responses to Acute Bouts of Aerobic Exercise, Mindfulness Meditation, and Combinations of Exercise and Meditation: a Randomized Controlled Intervention. https://doi.org/ 10.1177/0033294118755099

Engel, F.A., Rappelt, L., Held, S., & Donath, L. (2019) Can High-Intensity Functional Suspension Training over Eight Weeks Improve Resting Blood Pressure and Quality of Life in Young Adults? A Randomized Controlled Trial. *International journal of environmental research and public health*, *16*(24). https://doi.org/10.3390/ijerph16245062

Enrique Roig, A., Mooney, O., Salamanca-Sanabria, A., Lee, C.T., Farrell, S., & Richards, D. (2020) Assessing the Efficacy and Acceptability of a Web-Based Intervention for Resilience Among College Students: Pilot Randomized Controlled Trial. *JMIR formative research*, *4*(11), e20167. https://doi.org/10.2196/20167

Erogul, M., Singer, G., McIntyre, T., & Stefanov, D.G. (2014) Abridged mindfulness intervention to support wellness in first-year medical students. *Teach Learn Med*, *26*(4), 350-6. https://doi.org/10.1080/10401334.2014.945025

Fernández-Portero, C., Alarcón, D., Gallardo-Flores, A., Amián, J.G., & Sánchez-Medina, J.A. (2021) Effectiveness of a Mindfulness-Based Intervention Program for Women Family Caregivers of Older Adults. *Healthcare, 9*(9). https://doi.org/10.3390/healthcare9091216

Fisher, K., & Li, F. (2004) A community-based walking trial to improve neighborhood quality of life in older adults: A multilevel analysis. *Ann. Behav. Med.*, *28*(3), 186-194. https://doi.org/10.1207/s15324796abm2803_7

Flook, L., Goldberg, S.B., Pinger, L., Bonus, K., & Davidson, R.J. (2013) Mindfulness for Teachers: A Pilot Study to Assess Effects on Stress, Burnout, and Teaching Efficacy. *Mind, Brain, and Education*, *7*(3), 182-195. https://doi.org/https://doi.org/10.1111/mbe.12026

Galante, J., Bekkers, M., Mitchell, C., & Gallacher, J. (2016) Loving-Kindness Meditation Effects on Well-Being and Altruism: A Mixed-Methods Online RCT. *Applied psychology. Health and well-being*, *8*(3), 322-350. https://doi.org/10.1111/aphw.12074

Gerodimos, V., Karatrantou, K., Papazeti, K., Batatolis, C., & Krommidas, C. (2022) Workplace exercise program in a hospital environment: an effective strategy for the promotion of employees physical and mental health. A randomized controlled study. *Int. Arch. Occup. Environ. Health*. https://doi.org/10.1007/s00420-022-01856-6

Goldberg, S.B., Imhoff-Smith, T., Bolt, D.M., Wilson-Mendenhall, C.D., Dahl, C.J., Davidson, R.J., & Rosenkranz, M.A. (2020) Testing the Efficacy of a Multicomponent, Self-Guided, Smartphone-Based Meditation App: Three-Armed Randomized Controlled Trial. *JMIR mental health*, *7*(11), e23825. https://doi.org/10.2196/23825

Green, L.S., Oades, L.G., & Grant, A.M. (2006) Cognitive-behavioral, solution-focused life coaching: Enhancing goal striving, well-being, and hope. *The Journal of Positive Psychology*, *1*(3), 142-149. https://doi.org/10.1080/17439760600619849

Green, Z. (2022) Character strengths intervention for nurturing well-being among Pakistan's university students: a mixed-method study. https://doi.org/ 10.1111/aphw.12301

Gregoire, S., Lachance, L., Bouffard, T., & Dionne, F. (2017) The Use of Acceptance and Commitment Therapy to Promote Mental Health and School Engagement in University Students: a Multisite Randomized Controlled Trial. https://doi.org/ 10.1016/j.beth.2017.10.003

Hajatnia, B., Tajeri, B., & Alizadeh, K. (2021) Effectiveness of acceptance and commitment therapy in sleep quality, resilience, and death anxiety in the elderly. https://doi.org/ 10.30483/rijm.2021.254167.1021

Harris, A.R., Jennings, P.A., Katz, D.A., Abenavoli, R.M., & Greenberg, M.T. (2016) Promoting Stress Management and Wellbeing in Educators: Feasibility and Efficacy of a School-Based Yoga and Mindfulness Intervention. *Mindfulness*, *7*(1), 143-154. https://doi.org/10.1007/s12671-015-0451-2

Heintzelman, S., Kushlev, K., Lutes, L., Wirtz, D., Kanippayoor, J., Leitner, D., Oishi, S., & Diener, E. (2019) ENHANCE: Evidence for the Efficacy of a Comprehensive Intervention Program to Promote Subjective Well-Being. *J. Exp. Psychol. Appl.*. https://doi.org/10.1037/xap0000254

Hendriks, T., Schotanus-Dijkstra, M., Hassankhan, A., Sardjo, W., Graafsma, T., Bohlmeijer, E., & de Jong, J. (2020) Resilience and well-being in the Caribbean: Findings from a randomized controlled trial of a culturally adapted multi-component positive psychology intervention. *The Journal of Positive Psychology*, *15*(2), 238-253. https://doi.org/10.1080/17439760.2019.1590624

Hilcove, K., Marceau, C., Thekdi, P., Larkey, L., Brewer, M.A., & Jones, K. (2021) Holistic Nursing in Practice: Mindfulness-Based Yoga as an Intervention to Manage Stress and Burnout. *Journal of holistic nursing : official journal of the American Holistic Nurses' Association*, *39*(1), 29-42. https://doi.org/10.1177/0898010120921587

Hirshberg, M.J., Frye, C., Dahl, C.J., Riordan, K.M., Vack, N.J., Sachs, J., Goldman, R., Davidson, R.J., & Goldberg, S.B. (2022) A Randomized Controlled Trial of a Smartphone-Based Well-Being Training in Public School System Employees During the COVID-19 Pandemic. *Journal of educational psychology*, *114*(8), 1895-1911. https://doi.org/10.1037/edu0000739

Hirshberg, M.J., Goldberg, S.B., Schaefer, S.M., Flook, L., Findley, D., & Davidson, R.J. (2018) Divergent effects of brief contemplative practices in response to an acute stressor: A randomized controlled trial of brief breath awareness, loving-kindness, gratitude or an attention control practice. *PloS one*, *13*(12), e0207765. https://doi.org/10.1371/journal.pone.0207765

Ho, P., Li, T., Liu, H., Yeung, T., & Hou, W. (2022) Testing a New Protocol of Nature-Based Intervention to Enhance Well-Being: a Randomized Control Trial. https://doi.org/ 10.3390/ijerph19073931

Hollingsworth, J., & Redden, D. (2022) Tiny Habits® for Gratitude-Implications for Healthcare Education Stakeholders. https://doi.org/ 10.3389/fpubh.2022.866992

Hu, L., Zhu, L., Lyu, J., Zhu, W., Xu, Y., & Yang, L. (2017) Benefits of Walking on Menopausal Symptoms and Mental Health Outcomes among Chinese Postmenopausal Women. *Int. J. Gerontol.*, *11*(3), 166-170. https://doi.org/10.1016/j.ijge.2016.08.002

Huang, J., Lin, K., Fan, L., Qiao, S., & Wang, Y. (2021) The effects of a self-compassion intervention on future-oriented coping and psychological well-being: A randomized controlled trial in Chinese college students. *Mindfulness*, *12*(6), 1451-1458. https://doi.org/10.1007/s12671-021-01614-8

Hunsinger, M., Goerling, S., Bowen, C., Gross, J., Pruessner, M., & Christopher (2018) Mindfulness-based resilience training to reduce health risk, stress reactivity, and aggression among law enforcement officers: a feasibility and preliminary efficacy trial. https://doi.org/ 10.1177/2164956118773837

Hunt, M., Al-Braiki, F., Dailey, S., Russell, R., & Simon, K. (2018) Mindfulness Training, Yoga, or Both? Dismantling the Active Components of a Mindfulness-Based Stress Reduction Intervention. *Mindfulness*, *9*(2), 512-520. https://doi.org/10.1007/s12671-017-0793-z

Hwang, Y., Goldstein, H., Medvedev, O.N., Singh, N.N., Noh, J., & Hand, K. (2019) Mindfulness-Based Intervention for Educators: Effects of a School-Based Cluster Randomized Controlled Study. *Mindfulness*, *10*(7), 1417-1436. https://doi.org/10.1007/s12671-019-01147-1

Ivtzan, I., Young, T., Lee, H.C., Lomas, T., Daukantaitė, D., & Kjell, O.N.E. (2018) Mindfulness Based Flourishing Program: A cross-cultural study of Hong Kong Chinese and British participants. *Journal of Happiness Studies: An Interdisciplinary Forum on Subjective Well-Being*, *19*(8), 2205-2223. https://doi.org/10.1007/s10902-017-9919-1

Ivtzan, I., Young, T., Martman, J., Jeffrey, A., Lomas, T., Hart, R., & Eiroa-Orosa, F.J. (2016) Integrating mindfulness into positive psychology: A randomised controlled trial of an online Positive Mindfulness Program. *Mindfulness*, *7*(6), 1396-1407. https://doi.org/10.1007/s12671-016-0581-1

Janssen, M., Heerkens, Y., Van der Heijden, B., Korzilius, H., Peters, P., & Engels, J. (2022) Effects of mindfulness-based stress reduction and an organizational health intervention on Dutch teachers' mental health. https://doi.org/ 10.1093/heapro/daac008

Janzarik, G., Wollschläger, D., Wessa, M., & Lieb, K. (2022)A Group Intervention to Promote Resilience in Nursing Professionals: A Randomised Controlled Trial. *International journal of environmental research and public health*, *19*(2). https://doi.org/10.3390/ijerph19020649

Jarukasemthawee, S., Halford, W.K., & McLean, J.P. (2019) When East meets West: A randomized controlled trial and pre- to postprogram evaluation replication of the effects of insight-based mindfulness on psychological well-being. *Journal of Psychotherapy Integration*, *29*(3), 307-323. https://doi.org/10.1037/int0000179

Jazaieri, H., Jinpa, G.T., McGonigal, K., Rosenberg, E.L., Finkelstein, J., Simon-Thomas, E., Cullen, M., Doty, J.R., Gross, J.J., & Goldin, P.R. (2013) Enhancing compassion: A randomized controlled trial of a compassion cultivation training program. *Journal of Happiness Studies: An Interdisciplinary Forum on Subjective Well-Being*, *14*(4), 1113-1126. https://doi.org/10.1007/s10902-012-9373-z

Jazaieri, H., McGonigal, K., Jinpa, T., Doty, J.R., Gross, J.J., & Goldin, P.R. (2014) A randomized controlled trial of compassion cultivation training: Effects on mindfulness, affect, and emotion regulation. *Motivation and Emotion*, *38*(1), 23-35. https://doi.org/10.1007/s11031-013-9368-z

Jennings, P.A., Doyle, S., Oh, Y., Rasheed, D., Frank, J.L., & Brown, J.L. (2019) Long-term impacts of the CARE program on teachers' self-reported social and emotional competence and well-being. *Journal of school psychology*, *76*, 186-202. https://doi.org/10.1016/j.jsp.2019.07.009

Jones, D., Lehman, B., Noriega, A., & Dinnel, D. (2019) The effects of a short-term mindfulness meditation intervention on coping flexibility. https://doi.org/ 10.1080/10615806.2019.1596672

Josefsson, T., Lindwall, M., & Broberg, A.G. (2014) The effects of a short-term mindfulness-based intervention on self-reported mindfulness, decentering, executive attention, psychological health, and coping style: Examining unique mindfulness effects and mediators. *Mindfulness*, *5*(1), 18-35. https://doi.org/10.1007/s12671-012-0142-1

Juul, L., Brorsen, E., Gøtzsche, K., Nielsen, B.L., & Fjorback, L.O. (2021)The Effects of a Mindfulness Program on Mental Health in Students at an Undergraduate Program for Teacher Education: A Randomized Controlled Trial in Real-Life. *Frontiers in psychology*, *12*, 722771. https://doi.org/10.3389/fpsyg.2021.722771

Kadian, S., Joseph, J., Pal, S., & Devi, R. (2022) Brief resilience interventions for mental health among college students: Randomized controlled trial. *Asian. J. Soc. Health. Behav.*, *5*(3), 131-137. https://doi.org/10.4103/shb.shb-28-22

Kaemmerer, M., Congard, A., Le Vigouroux, S., Dauvier, B., Andreotti, E., & Antoine, P. (2022) Do Mindfulness-Based Interventions Have Effects Only on Negative Aspects of Psychological Functioning? A Randomized Controlled Trial. *Mindfulness*, *13*(5), 1158-1172. https://doi.org/10.1007/s12671-022-01849-z

Kam, J., Javed, J., Hart, C., Andrews-Hanna, J., Tomfohr-Madsen, L., & Mills, C. (2022) Daily mindfulness training reduces negative impact of COVID-19 news exposure on affective well-being. https://doi.org/ 10.1007/s00426-021-01550-1

Kamegaya, T., Araki, Y., Kigure, H., & Yamaguchi, H. (2014) Twelve-week physical and leisure activity programme improved cognitive function in community-dwelling elderly subjects: A randomized controlled trial. *Psychogeriatrics*, *14*(1), 47-54. https://doi.org/10.1111/psyg.12038

Kang, M.Y., Nan, J.K.M., & Yuan, Y. (2022) Effects and mechanisms of an online short-term audio-based mindfulness program on positive affect: A randomized controlled trial including exploratory moderator analyses. *Applied psychology. Health and well-being*. https://doi.org/10.1111/aphw.12431

Karing, C., & Beelmann, A. (2021). Evaluating the implementation and effectiveness of a low-dose mindfulness-based intervention in a student sample: a randomized controlled trial. *Mindfulness*, *12*, 1438-1450. https://doi.org/10.1007/s12671-021-01613-9

Kariyawasam, L., Ononaiye, M., Irons, C., & Kirby, S.E. (2022) Exploring the Cross-cultural Applicability of a Brief Compassionate Mind Training: a Study Comparing Sri Lankan and UK People. *Mindfulness*, 1-19. https://doi.org/10.1007/s12671-022-02041-z

Khazaee-Pool, M., Sadeghi, R., Majlessi, F., & Rahimi Foroushani, A. (2015) Effects of physical exercise programme on happiness among older people. *Journal of psychiatric and mental health nursing*, *22*(1), 47-57. https://doi.org/10.1111/jpm.12168

Kimura, K., Obuchi, S., Arai, T., Nagasawa, H., Shiba, Y., Watanabe, S., & Kojima, M. (2010) The influence of short-term strength training on health-related quality of life and executive cognitive function. https://doi.org/ 10.2114/jpa2.29.95

Kloos, N., Austin, J., van 't Klooster, J., Drossaert, C., & Bohlmeijer, E. (2022) Appreciating the Good Things in Life During the Covid-19 Pandemic: A Randomized Controlled Trial and Evaluation of a Gratitude App. *Journal of happiness studies*, *23*(8), 4001-4025. https://doi.org/10.1007/s10902-022-00586-3

Ko, C.M., Grace, F., Chavez, G.N., Grimley, S.J., Dalrymple, E.R., & Olson, L.E. (2018) Effect of Seminar on Compassion on student self-compassion, mindfulness and well-being: A randomized controlled trial. *Journal of American college health: J of ACH*, *66*(7), 537-545. https://doi.org/10.1080/07448481.2018.1431913

Kosugi, T., Ninomiya, A., Nagaoka, M., Hashimoto, Z., Sawada, K., Park, S., Fujisawa, D., Mimura, M., & Sado, M. (2021) Effectiveness of Mindfulness-Based Cognitive Therapy for Improving Subjective and Eudaimonic Well-Being in Healthy Individuals: A Randomized Controlled Trial. *Frontiers in psychology*, *12*, 700916. https://doi.org/10.3389/fpsyg.2021.700916

Krekel, C., De Neve, J., Fancourt, D., & Layard, R. (2021) A local community course that raises wellbeing and pro-sociality: Evidence from a randomised controlled trial. *Journal of Economic Behavior & Organization*, *188*, 322-336. https://doi.org/10.1016/j.jebo.2021.05.021

Kuhlthau, K., Luberto, C., Traeger, L., Millstein, R., Perez, G., Lindly, O., Chad-Friedman, E., Proszynski, J., & Park, E. (2020) A Virtual Resiliency Intervention for Parents of Children with Autism: A Randomized Pilot Trial. *J. Autism Dev. Disord.*, *50*(7), 2513-2526. https://doi.org/10.1007/s10803-019-03976-4

Kwon, S.H. (2015) Wheel of Wellness Counseling in Community Dwelling, Korean Elders: A Randomized, Controlled Trial. *Journal of Korean Academy of Nursing*, *45*(3), 459-468. https://doi.org/10.4040/jkan.2015.45.3.459

LeBlanc, S., Uzun, B., Pourseied, K., & Mohiyeddini, C. (2017) Effect of an Emotion Regulation Training Program on Mental Well-Being. *International Journal of Group Psychotherapy*, *67*(1), 108-123. https://doi.org/10.1080/00207284.2016.1203585

Lee, A.S., McInnes, R.J., Hughes, A.R., Guthrie, W., & Jepson, R. (2016) The Effect of the More Active MuMs in Stirling Trial on Body Composition and Psychological Well-Being among Postnatal Women. *Journal of pregnancy*, *2016*, 4183648. https://doi.org/10.1155/2016/4183648

Lee, T. S. H., Hung, C. C., Lin, C. K., & Chiang, H. H. (2019). Controlled randomized trial of walking exercise with positive education on cardiovascular fitness and happiness in retired older adults. *Geriatrics & gerontology international*, *19*(9), 879-884. https://doi.org/10.1111/ggi.13733

Lee, E., Wong, B., Chan, P., Zhang, D., Sun, W., Chan, D., Gao, T., Ho, F., Kwok, T., & Wong, S. (2021) Effectiveness of a mindfulness intervention for older adults to improve emotional well-being and cognitive function in a Chinese population: a randomized waitlist-controlled trial. https://doi.org/ 10.1002/gps.5616

Li, F., Duncan, T.E., Duncan, S.C., McAuley, E., Chaumeton, N.R., & Harmer, P. (2001) Enhancing the psychological well-being of elderly individuals through Tai Chi exercise: A latent growth curve analysis. *Structural Equation Modeling*, *8*(1), 53-83. https://doi.org/10.1207/S15328007SEM0801_4

Li, M., Fang, Q., Li, J., Zheng, X., Tao, J., Yan, X., Lin, Q., Lan, X., Chen, B., Zheng, G., & Chen, L. (2015) The Effect of Chinese Traditional Exercise-Baduanjin on Physical and Psychological Well-Being of College Students: A Randomized Controlled Trial. *PloS one*, *10*(7), e0130544. https://doi.org/10.1371/journal.pone.0130544

Liu, C., Chen, H., Liang, Y., Hsu, S., Huang, D., Liu, C., & Chiou, W. (2022) The effect of loving-kindness meditation on employees' mindfulness, affect, altruism and knowledge hiding. *BMC psychology*, *10*(1), 138. https://doi.org/10.1186/s40359-022-00846-0

Liu, C., Chen, H., Liu, C., Lin, R., & Chiou, W. (2020) The Effect of Loving-Kindness Meditation on Flight Attendants' Spirituality, Mindfulness and Subjective Well-Being. *Healthcare*, *8*(2), 174.

Liu, J., Zhang, Y., Li, X., Wang, D., Shi, B., You, Y., Min, L., Luo, B., Li, Y., Di, Q., & Ma, X. (2022) Exercise improves mental health status of young adults via attenuating inflammation factors but modalities matter. *Frontiers in psychiatry*, *13*, 1067890. https://doi.org/10.3389/fpsyt.2022.1067890

Loewenthal, J., Dyer, N.L., Lipsyc-Sharf, M., Borden, S., Mehta, D.H., Dusek, J.A., & Khalsa, S.B.S. (2021) Evaluation of a Yoga-Based Mind-Body Intervention for Resident Physicians: A Randomized Clinical Trial. *Global advances in health and medicine*, *10*, 21649561211001038. https://doi.org/10.1177/21649561211001038

Lorenz, T., Algner, M., & Binder, B. (2022) A Positive Psychology Resource for Students? Evaluation of the Effectiveness of the 6 Minutes Diary in a Randomized Control Trial. *Frontiers in psychology*, *13*, 896741. https://doi.org/10.3389/fpsyg.2022.896741

Ly, K.H., Ly, A., & Andersson, G. (2017) A fully automated conversational agent for promoting mental well-being: A pilot RCT using mixed methods. *Internet interventions*, *10*, 39-46. https://doi.org/10.1016/j.invent.2017.10.002

Maatouk, I., Müller, A., Angerer, P., Schmook, R., Nikendei, C., Herbst, K., Gantner, M., Herzog, W., & Gündel, H. (2018) Healthy ageing at work- Efficacy of group interventions on the mental health of nurses aged 45 and older: Results of a randomised, controlled trial. *PloS one*, *13*(1), e0191000. https://doi.org/10.1371/journal.pone.0191000

Mackenzie, C., Poulin, P., & Seidman-Carlson, R. (2006) A brief mindfulness-based stress reduction intervention for nurses and nurse aides. *Appl. Nurs. Res.*, *19*(2), 105-109. https://doi.org/10.1016/j.apnr.2005.08.002

Mak, W.W.S., Chan, A.T.Y., Cheung, E.Y.L., Lin, C.L.Y., & Ngai, K.C.S. (2015) Enhancing Web-based mindfulness training for mental health promotion with the health action process approach: randomized controlled trial. *Journal of medical Internet research*, *17*(1), e8. https://doi.org/10.2196/jmir.3746

Manotas, M., Segura, C., Eraso, M., Oggins, J., & McGovern, K. (2014) Association of brief mindfulness training with reductions in perceived stress and distress in Colombian health care professionals. *International Journal of Stress Management*, *21*, 207-225. https://doi.org/10.1037/a0035150

Mascaro, J.S., Palmer, P.K., Ash, M.J., Peacock, C., Sharma, A., Escoffery, C., & Raison, C. (2021) Feasibility, Acceptability, and Preliminary Effectiveness of a Compassion-Centered Team Intervention to Improve Clinical Research Coordinator Resilience and Well-Being. *JCO oncology practice*, *17*(7), e936-e946. https://doi.org/10.1200/OP.21.00120

Matos, M., Albuquerque, I., Galhardo, A., Cunha, M., Pedroso Lima, M., Palmeira, L., Petrocchi, N., McEwan, K., Maratos, F.A., & Gilbert, P. (2022) Nurturing compassion in schools: A randomized controlled trial of the effectiveness of a Compassionate Mind Training program for teachers. *PloS one*, *17*(3), e0263480. https://doi.org/10.1371/journal.pone.0263480

Matvienko-Sikar, K., & Dockray, S. (2017) Effects of a novel positive psychological intervention on prenatal stress and well-being: A pilot randomised controlled trial. *Women Birth*, *30*(2), e111-e118. https://doi.org/10.1016/j.wombi.2016.10.003

Maurer, A., Deckert, S., Levenig, C., Schörkmaier, T., Stangier, C., Attenberger, U., Hasenbring, M., & Boecker, H. (2020) Body Image Relates to Exercise-Induced Antinociception and Mood Changes in Young Adults: a Randomized Longitudinal Exercise Intervention. https://doi.org/ 10.3390/ijerph17186801

McConachie, D., McKenzie, K., Morris, P., & Walley, R. (2014) Acceptance and mindfulness-based stress management for support staff caring for individuals with intellectual disabilities. https://doi.org/ 10.1016/j.ridd.2014.03.005

McGonagle, A.K., Schwab, L., Yahanda, N., Duskey, H., Gertz, N., Prior, L., Roy, M., & Kriegel, G. (2020) Coaching for primary care physician well-being: A randomized trial and follow-up analysis. *Journal of occupational health psychology*, *25*(5), 297-314. https://doi.org/10.1037/ocp0000180

Miller, R.W., & Duncan, E. (2015) A pilot randomised controlled trial comparing two positive psychology interventions for their capacity to increase subjective wellbeing. *Counselling Psychology Review*, *30*(3), 36-46.

Mirabito, G., & Verhaeghen, P. (2022) Remote delivery of a Koru Mindfulness intervention for college students during the COVID-19 pandemic. *Journal of American college health: J of ACH*, 1-8. https://doi.org/10.1080/07448481.2022.2060708

Montaner, X., Tárrega, S., Pulgarin, M., & Moix, J. (2022) Effectiveness of Acceptance and Commitment Therapy (ACT) in Professional Dementia Caregivers Burnout. https://doi.org/ 10.1080/07317115.2021.1920530

Morledge, T.J., Allexandre, D., Fox, E., Fu, A.Z., Higashi, M.K., Kruzikas, D.T., Pham, S.V., & Reese, P.R. (2013) Feasibility of an online mindfulness program for stress management--a randomized, controlled trial. *Annals of behavioral medicine : a publication of the Society of Behavioral Medicine*, *46*(2), 137-148. https://doi.org/10.1007/s12160-013-9490-x

Mountain, G., Windle, G., Hind, D., Walters, S., Keertharuth, A., Chatters, R., Sprange, K., Craig, C., Cook, S., Lee, E., Chater, T., Woods, R., Newbould, L., Powell, L., Shortland, K., & Roberts, J. (2017) A preventative lifestyle intervention for older adults (lifestyle matters): a randomised controlled trial. *Age and ageing*, *46*(4), 627-634. https://doi.org/10.1093/ageing/afx021

Müller, A., Heiden, B., Herbig, B., Poppe, F., & Angerer, P. (2016) Improving well-being at work: A randomized controlled intervention based on selection, optimization, and compensation. *Journal of Occupational Health Psychology*, *21*(2), 169.

Murray, K., Hellier Villafana, V., Sheik Mohamed, A., Linke, S., Bowen, D., & Marcus, B. (2021) Testing the feasibility and acceptability of a culturally adapted physical activity intervention for adult Somali women. https://doi.org/ 10.1093/tbm/ibab064

Myers, N.D., Prilleltensky, I., Prilleltensky, O., McMahon, A., Dietz, S., & Rubenstein, C.L. (2017) Efficacy of the Fun For Wellness Online Intervention to Promote Multidimensional Well-Being: a Randomized Controlled Trial. *Prevention science: the official journal of the Society for Prevention Research*, *18*(8), 984-994. https://doi.org/10.1007/s11121-017-0779-z

Nadler, R., Carswell, J.J., & Minda, J.P. (2020) Online Mindfulness Training Increases Well-Being, Trait Emotional Intelligence, and Workplace Competency Ratings: A Randomized Waitlist-Controlled Trial. *Front Psychol*, *11*, 255. https://doi.org/10.3389/fpsyg.2020.00255

Nedeljkovic, M., Wirtz, P.H., & Ausfeld-Hafter, B. (2012) Effects of Taiji practice on mindfulness and self-compassion in healthy participants—A randomized controlled trial. *Mindfulness*, *3*(3), 200-208. https://doi.org/10.1007/s12671-012-0092-7

Neff, K.D., & Germer, C.K. (2013) A pilot study and randomized controlled trial of the mindful self-compassion program. *Journal of clinical psychology*, *69*(1), 28-44. https://doi.org/10.1002/jclp.21923

Neumeier, L.M., Brook, L., Ditchburn, G., & Sckopke, P. (2017) Delivering your daily dose of well-being to the workplace: A randomized controlled trial of an online well-being programme for employees. *European Journal of Work and Organizational Psychology*, *26*(4), 555-573. https://doi.org/10.1080/1359432X.2017.1320281

Ng, K., Chan, H., Sia, A., Mahendran, R., Tan, C., Feng, L., Kian-Wee Ng, M., Tan, C., Larbi, A., Ho, R., & et al. (2016) The effects of horticultural therapy on the psychological well-being and associated biomarkers of elderly in Singapore.

Nguyen, H.V., Huang, H., Wong, M., Yang, Y., Huang, T., & Teng, C. (2018) Moderator Roles of Optimism and Weight Control on the Impact of Playing Exergames on Happiness: The Perspective of Social Cognitive Theory Using a Randomized Controlled Trial. *Games for health journal*, *7*(4), 246-252. https://doi.org/10.1089/g4h.2017.0165

Nielsen, E., & Minda, J. (2021) The Mindful Lawyer: investigating the Effects of Two Online Mindfulness Programs on Self-Reported Well-Being in the Legal Profession. https://doi.org/ 10.1097/JOM.0000000000002393

Noradechanunt, C., Worsley, A., & Groeller, H. (2017) Thai Yoga improves physical function and well-being in older adults: A randomised controlled trial. *Journal of science and medicine in sport*, *20*(5), 494-501. https://doi.org/10.1016/j.jsams.2016.10.007

Norman, E., Sherburn, M., Osborne, R.H., & Galea, M.P. (2010) An exercise and education program improves well-being of new mothers: a randomized controlled trial. *Physical therapy*, *90*(3), 348-355. https://doi.org/10.2522/ptj.20090139

O'Leary, K., & Dockray, S. (2015). The effects of two novel gratitude and mindfulness interventions on well-being. *The Journal of Alternative and Complementary Medicine*, *21*(4), 243-245.

Oken, B.S., Zajdel, D., Kishiyama, S., Flegal, K., Dehen, C., Haas, M., Kraemer, D.F., Lawrence, J., & Leyva, J. (2006) Randomized, controlled, six-month trial of yoga in healthy seniors: effects on cognition and quality of life. *Alternative therapies in health and medicine*, *12*(1), 40-47.

Oliver, J. J., & MacLeod, A. K. (2018). Working adults' well-being: An online self-help goal-based intervention. *Journal of Occupational and Organizational Psychology*, *91*(3), 665-680. https://doi.org/10.1111/joop.12212

Pacanowski, C. R., Diers, L., Crosby, R. D., Mackenzie, M., & Neumark-Sztainer, D. (2022). Yoga's impact on risk and protective factors for disordered eating: a pilot prevention trial. In *Yoga for Positive Embodiment in Eating Disorder Prevention and Treatment* (pp. 210-238). Routledge.

Page, K.M., & Vella-Brodrick, D.A. (2013) The working for wellness program: RCT of an employee well-being intervention. *Journal of Happiness Studies: An Interdisciplinary Forum on Subjective Well-Being*, *14*(3), 1007-1031. https://doi.org/10.1007/s10902-012-9366-y

Pan, W., Chang, C., Chen, S., & Gau, M. (2019) Assessing the effectiveness of mindfulness-based programs on mental health during pregnancy and early motherhood - a randomized control trial. https://doi.org/ 10.1186/s12884-019-2503-4

Pang, D., & Ruch, W. (2019). Fusing character strengths and mindfulness interventions: Benefits for job satisfaction and performance. *Journal of occupational health psychology*, *24*(1), 150.

Passmore, H., Yargeau, A., & Blench, J. (2022) Wellbeing in Winter: Testing the Noticing Nature Intervention During Winter Months. *Frontiers in psychology*, *13*, 840273. https://doi.org/10.3389/fpsyg.2022.840273

Payne, E., Loi, N., & Thorsteinsson, E. (2020) The Restorative Effect of the Natural Environment on University Students' Psychological Health. *J. Environ. Public Health*, *2020*. https://doi.org/10.1155/2020/4210285

Perez-Blasco, J., Sales, A., Meléndez, J.C., & Mayordomo, T. (2016) The Effects of Mindfulness and Self-Compassion on Improving the Capacity to Adapt to Stress Situations in Elderly People Living in the Community. *Clinical Gerontologist*, *39*(2), 90-103. https://doi.org/10.1080/07317115.2015.1120253

Proyer, R., Gander, F., Wellenzohn, S., & Ruch, W. (2016) Addressing the role of personality, ability, and positive and negative affect in positive psychology interventions: Findings from a randomized intervention based on the authentic happiness theory and extensions. *J. Posit. Psychol.*, *11*(6), 609-621. https://doi.org/10.1080/17439760.2015.1137622

Rababah, J., & Al-Hammouri, M. (2022) Effect of a modified motivational interviewing intervention on university students' psychological, cognitive, and nutritional health: a randomized controlled trial. https://doi.org/ 10.1111/nuf.12841

Read, A., Mazzucchelli, T.G., & Kane, R.T. (2016) A preliminary evaluation of a single session behavioural activation intervention to improve well-being and prevent depression in carers. *Clinical Psychologist*, *20*(1), 36-45.

Recabarren, R.E., Gaillard, C., Guillod, M., & Martin-Soelch, C. (2019) Short-Term Effects of a Multidimensional Stress Prevention Program on Quality of Life, Well-Being and Psychological Resources. A Randomized Controlled Trial. *Frontiers in psychiatry*, *10*, 88. https://doi.org/10.3389/fpsyt.2019.00088

Rich, R.M., Ogden, J., & Morison, L. (2021) A randomized controlled trial of an app-delivered mindfulness program among university employees: effects on stress and work-related outcomes. *International Journal of Workplace Health Management*, *14*(2), 201-216. https://doi.org/10.1108/IJWHM-04-2020-0046

Rodríguez-Jiménez, R., Carmona, M., García-Merino, S., Díaz-Rivas, B., & Thuissard-Vasallo, I. (2022) Stress, subjective wellbeing and selfknowledge in higher education teachers: A pilot study through bodyfulness approaches. *PLoS ONE*, *17*(12 December). https://doi.org/10.1371/journal.pone.0278372

Roeser, R.W., Schonert-Reichl, K.A., Jha, A., Cullen, M., Wallace, L., Wilensky, R., Oberle, E., Thomson, K., Taylor, C., & Harrison, J. (2013) Mindfulness training and reductions in teacher stress and burnout: Results from two randomized, waitlist-control field trials. *Journal of Educational Psychology*, *105*(3), 787-804. https://doi.org/10.1037/a0032093

Santos, L., do Rosário Pinheiro, M., & Rijo, D. (2022) Compassionate mind training for caregivers of residential youth care: Early findings of a cluster randomized trial. *Child Abuse Negl.*, *123*. https://doi.org/10.1016/j.chiabu.2021.105429

Schulte-Frankenfeld, P.M., & Trautwein, F. (2022)App-based mindfulness meditation reduces perceived stress and improves self-regulation in working university students: A randomised controlled trial. *Applied psychology. Health and well-being*, *14*(4), 1151-1171. https://doi.org/10.1111/aphw.12328

Seear, K.H., & Vella-Brodrick, D.A. (2013) Efficacy of positive psychology interventions to increase well-being: Examining the role of dispositional mindfulness. *Social Indicators Research*, *114*(3), 1125-1141. https://doi.org/10.1007/s11205-012-0193-7

Senf, K., & Liau, A.K. (2013) The Effects of Positive Interventions on Happiness and Depressive Symptoms, with an Examination of Personality as a Moderator. *Journal of Happiness Studies*, *14*(2), 591-612. https://doi.org/10.1007/s10902-012-9344-4

Seppälä, E.M., Bradley, C., Moeller, J., Harouni, L., Nandamudi, D., & Brackett, M.A. (2020)Promoting Mental Health and Psychological Thriving in University Students: A Randomized Controlled Trial of Three Well-Being Interventions. *Frontiers in psychiatry*, *11*, 590. https://doi.org/10.3389/fpsyt.2020.00590

Shapiro, S.L., Brown, K.W., Thoresen, C., & Plante, T.G. (2011)The moderation of Mindfulness-based stress reduction effects by trait mindfulness: results from a randomized controlled trial. *Journal of clinical psychology*, *67*(3), 267-277. https://doi.org/10.1002/jclp.20761

Sommers-Spijkerman, M., Trompetter, H., Schreurs, K., & Bohlmeijer, E. (2018)Pathways to Improving Mental Health in Compassion-Focused Therapy: Self-Reassurance, Self-Criticism and Affect as Mediators of Change. *Frontiers in psychology*, *9*, 2442. https://doi.org/10.3389/fpsyg.2018.02442

Sood, A., Sharma, V., Schroeder, D.R., & Gorman, B. (2014)Stress Management and Resiliency Training (SMART) program among Department of Radiology faculty: a pilot randomized clinical trial. *Explore (New York, N.Y.)*, *10*(6), 358-363. https://doi.org/10.1016/j.explore.2014.08.002

Spence, G.B., & Cavanagh, M.J. (2019) The impact of three forms of mindfulness training on mindfulness, wellbeing and goal attainment: Findings from a randomised controlled trial and implications for coaching. *International Coaching Psychology Review*, *14*(2), 24-43.

Spence, G.B., & Grant, A.M. (2007) Professional and peer life coaching and the enhancement of goal striving and well-being: An exploratory study. *The Journal of Positive Psychology*, *2*(3), 185-194. https://doi.org/10.1080/17439760701228896

Steinhardt, M., & Dolbier, C. (2008) Evaluation of a resilience intervention to enhance coping strategies and protective factors and decrease symptomatology. *J. Am. Coll. Health*, *56*(4), 445-453. https://doi.org/10.3200/JACH.56.44.445-454

Strauss, C., Gu, J., Montero-Marin, J., Whittington, A., Chapman, C., & Kuyken, W. (2021)Reducing stress and promoting well-being in healthcare workers using mindfulness-based cognitive therapy for life. *International journal of clinical and health psychology : IJCHP*, *21*(2), 100227. https://doi.org/10.1016/j.ijchp.2021.100227

Sturm, V., Datta, S., Roy, A., Sible, I., Kosik, E., Veziris, C., Chow, T., Morris, N., Neuhaus, J., Kramer, J., Miller, B., Holley, S., & Keltner, D. (2022) Big Smile, Small Self: Awe Walks Promote Prosocial Positive Emotions in Older Adults. *Emotion*, *22*(5), 1044-1058. https://doi.org/10.1037/emo0000876

Taylor, H., Cavanagh, K., Field, A.P., & Strauss, C. (2022)Health Care Workers' Need for Headspace: Findings From a Multisite Definitive Randomized Controlled Trial of an Unguided Digital Mindfulness-Based Self-help App to Reduce Healthcare Worker Stress. *JMIR mHealth and uHealth*, *10*(8), e31744. https://doi.org/10.2196/31744

Taylor, B. L., Strauss, C., Cavanagh, K., & Jones, F. (2014). The effectiveness of self-help mindfulness-based cognitive therapy in a student sample: a randomised controlled trial. *Behaviour Research and Therapy*, *63*, 63-69. https://doi.org/10.1016/j.brat.2014.09.007

Terblanche, N., Molyn, J., De Haan, E., & Nilsson, V. (2022) Coaching at Scale: Investigating theEfficacy of Artificial Intelligence Coaching. *Int. J. Evid. Based Coach. Mentor.*, *20*(2), 20-36. https://doi.org/10.24384/5cgf-ab69

Timlin, D., & Simpson, E.E.A. (2017) A preliminary randomised control trial of the effects of Dru yoga on psychological well-being in Northern Irish first time mothers. *Midwifery*, *46*, 29-36. https://doi.org/https://doi.org/10.1016/j.midw.2017.01.005

Trombka, M., Demarzo, M., Campos, D., Antonio, S.B., Cicuto, K., Walcher, A.L., García-Campayo, J., Schuman-Olivier, Z., & Rocha, N.S. (2021)Mindfulness Training Improves Quality of Life and Reduces Depression and Anxiety Symptoms Among Police Officers: Results From the POLICE Study-A Multicenter Randomized Controlled Trial. *Frontiers in psychiatry*, *12*, 624876. https://doi.org/10.3389/fpsyt.2021.624876

Tsang, K.K.Y., Shum, K.K., Chan, W.W.L., Li, S.X., Kwan, H.W., Su, M.R., Wong, B.P.H., & Lam, S. (2021)Effectiveness and Mechanisms of Mindfulness Training for School Teachers in Difficult Times: A Randomized Controlled Trial. *Mindfulness*, *12*(11), 2820-2831. https://doi.org/10.1007/s12671-021-01750-1

van Dijk, I., Lucassen, P.L.B.J., Akkermans, R.P., van Engelen, B.G.M., van Weel, C., & Speckens, A.E.M. (2017)Effects of Mindfulness-Based Stress Reduction on the Mental Health of Clinical Clerkship Students: A Cluster-Randomized Controlled Trial. *Academic medicine : journal of the Association of American Medical Colleges*, *92*(7), 1012-1021. https://doi.org/10.1097/ACM.0000000000001546

van Emmerik, A.A.P., Berings, F., & Lancee, J. (2018) Efficacy of a Mindfulness-Based Mobile Application: a Randomized Waiting-List Controlled Trial. *Mindfulness*, *9*(1), 187-198. https://doi.org/10.1007/s12671-017-0761-7

Van Roie, E., Martien, S., Hurkmans, E., Pelssers, J., Seghers, J., Boen, F., & Delecluse, C.Ergometer-cycling with strict versus minimal contact supervision among the oldest adults: A cluster-randomised controlled trial. *Archives of gerontology and geriatrics*, *70*, 112-122. https://doi.org/10.1016/j.archger.2017.01.010

Verweij, H., van Ravesteijn, H., van Hooff, M.L.M., Lagro-Janssen, A.L.M., & Speckens, A.E.M. (2018) Mindfulness-Based Stress Reduction for Residents: A Randomized Controlled Trial. *Journal of General Internal Medicine*, *33*(4), 429-436. https://doi.org/10.1007/s11606-017-4249-x

Viskovich, S., & Pakenham, K.I. (2020)Randomized controlled trial of a web-based Acceptance and Commitment Therapy (ACT) program to promote mental health in university students. *Journal of clinical psychology*, *76*(6), 929-951. https://doi.org/10.1002/jclp.22848

Wadhen, V., & Cartwright, T. (2021). Feasibility and outcome of an online streamed yoga intervention on stress and wellbeing of people working from home during COVID-19. *Work*, *69*(2), 331-349. DOI: 10.3233/WOR-205325

Waelde, L.C., Meyer, H., Thompson, J.M., Thompson, L., & Gallagher-Thompson, D. (2017)Randomized Controlled Trial of Inner Resources Meditation for Family Dementia Caregivers. *Journal of clinical psychology*, *73*(12), 1629-1641. https://doi.org/10.1002/jclp.22470

Wang, F., & Boros, S. (2020) Effects of a pedometer-based walking intervention on young adults' sleep quality, stress and life satisfaction: Randomized controlled trial. *J. Bodywork Mov. Ther.*, *24*(4), 286-292. https://doi.org/10.1016/j.jbmt.2020.07.011

Welford, P., Östh, J., Hoy, S., Diwan, V., & Hallgren, M. (2022) Effects of yoga and aerobic exercise on wellbeing in physically inactive older adults: Randomized controlled trial (FitForAge). *Complementary therapies in medicine*, *66*, 102815. https://doi.org/10.1016/j.ctim.2022.102815

Weytens, F., Luminet, O., Verhofstadt, L., & Mikolajczak, M. (2014) An integrative theory-driven positive emotion regulation intervention. *PLoS ONE*, *9*(4). https://doi.org/10.1371/journal.pone.0095677

Wilke, J., Mohr, L., Yuki, G., Bhundoo, A.K., Jiménez-Pavón, D., Laiño, F., Murphy, N., Novak, B., Nuccio, S., Ortega-Gómez, S., Pillay, J.D., Richter, F., Rum, L., Sanchez-Ramírez, C., Url, D., Vogt, L., & Hespanhol, L. (2022)Train at home, but not alone: a randomised controlled multicentre trial assessing the effects of live-streamed tele-exercise during COVID-19-related lockdowns. *British journal of sports medicine*, *56*(12), 667-675. https://doi.org/10.1136/bjsports-2021-104994

Wingert, J.R., Jones, J.C., Swoap, R.A., & Wingert, H.M. (2022) Mindfulness-based strengths practice improves well-being and retention in undergraduates: a preliminary randomized controlled trial. *Journal of American college health : J of ACH*, *70*(3), 783-790. https://doi.org/10.1080/07448481.2020.1764005

Xiong, Y., Prasath, P.R., Zhang, Q., & Jeon, L. (2022) A mindfulness-based well-being group for international students in higher education: A pilot study. *Journal of Counseling & Development*, *100*(4), 374-385. https://doi.org/10.1002/jcad.12432

Xu, H., Eley, R., Kynoch, K., & Tuckett, A. (2021) Effects of mobile mindfulness on emergency department work stress: a randomised controlled trial. https://doi.org/ 10.1111/1742-6723.13836

Yang, E., Schamber, E., Meyer, R.M.L., & Gold, J.I. (2018) Happier Healers: Randomized Controlled Trial of Mobile Mindfulness for Stress Management. *Journal of alternative and complementary medicine (New York, N.Y.)*, *24*(5), 505-513. https://doi.org/10.1089/acm.2015.0301

Yıldırım, D., & Çiriş Yıldız, C.The Effect of Mindfulness-Based Breathing and Music Therapy Practice on Nurses' Stress, Work-Related Strain, and Psychological Well-being During the COVID-19 Pandemic: A Randomized Controlled Trial. *Holistic nursing practice*, *36*(3), 156-165. https://doi.org/10.1097/HNP.0000000000000511

Zeng, X., Wang, R., Oei, T.P.S., & Leung, F.Y.K. (2019) Heart of Joy: a Randomized Controlled Trail Evaluating the Effect of an Appreciative Joy Meditation Training on Subjective Well-Being and Attitudes. *Mindfulness*, *10*(3), 506-515. https://doi.org/10.1007/s12671-018-0992-2

Zhang, Q., Wang, Z., Wang, X., Liu, L., Zhang, J., & Zhou, R. (2019) The effects of different stages of mindfulness meditation training on emotion regulation. *Front. Human Neurosci.*, *13*. https://doi.org/10.3389/fnhum.2019.00208

Zheng, Y., Zhou, J., Zeng, X., Jiang, M., & Oei, T. (2022) A New Second-Generation Mindfulness-Based Intervention Focusing on Well-Being: A Randomized Control Trial of Mindfulness-Based Positive Psychology. *J. Happiness Stud.*, *23*(6), 2703-2724. https://doi.org/10.1007/s10902-022-00525-2

Zilcha-Mano, S., & Langer, E. (2016) Mindful Attention to Variability Intervention and Successful Pregnancy Outcomes. *Journal of Clinical Psychology*, *72*(9), 897-907. https://doi.org/https://doi.org/10.1002/jclp.22294

1.4. Cochrane risk of bias for individual studies using RoB 2 (completed before node adaption/exclusion)

Study Name	1.0 Algorithm Result	2.0 Algorithm result	3.0 Algorithm Result	4.0 Algortithim Result	5.0 Algorithim Result	Overall
Aeschbach 2022	Low Risk	Low Risk	Low Risk	Low Risk	Some Concerns	Some Concerns
Ahmad 2020	Low Risk	Low Risk	Low Risk	Low Risk	Some Concerns	Some Concerns
Aikens 2014	Low Risk	Some Concerns	Low Risk	Low Risk	Some Concerns	Some Concerns
Ajilchi 2019	Low Risk	Some Concerns	Low Risk	Low Risk	Some Concerns	Some Concerns
Alexander 2015	Some Concerns	Some Concerns	Low Risk	Low Risk	Some Concerns	High Risk
Allexandre 2016	Low Risk	Some Concerns	Low Risk	Low Risk	Low Risk	Some Concerns
Amutio 2015	Low Risk	Some Concerns	Low Risk	Low Risk	Some Concerns	Some Concerns
Ardi 2021	Low Risk	Some Concerns	Low Risk	Low Risk	Some Concerns	Some Concerns
Asuero 2014	Low Risk	Some Concerns	Low Risk	Low Risk	Some Concerns	Some Concerns
Baker 2008	Low Risk	Some Concerns	Low Risk	Low Risk	Some Concerns	Some Concerns
Basso 2022	Low Risk	Some Concerns	Low Risk	Low Risk	Some Concerns	Some Concerns
Bita 2021	Low Risk	Some Concerns	Low Risk	Low Risk	Some Concerns	Some Concerns
Blasche 2013	Low Risk	High Risk	Low Risk	Low Risk	Some Concerns	High Risk
Bohlmeijer 2021	Low Risk	High Risk	Low Risk	Low Risk	Low Risk	High Risk
Bonde 2022	Low Risk	Some Concerns	Low Risk	Low Risk	Some Concerns	Some Concerns
Borchardt 2018	Low Risk	High Risk	Low Risk	Low Risk	Some Concerns	High Risk
Bowden 2011	Low Risk	High Risk	High Risk	Low Risk	Some Concerns	High Risk
Brinkmann 2020	Low Risk	High Risk	Some Concerns	Low Risk	Some Concerns	High Risk
Brito-Pons 2018	Low Risk	Some Concerns	Low Risk	Low Risk	Some Concerns	Some Concerns
Cerna 2020	Low Risk	High Risk	High Risk	Low Risk	Some Concerns	High Risk

Study Name	1.0 Algorithm Result	2.0 Algorithm result	3.0 Algorithm Result	4.0 Algortithim Result	5.0 Algorithim Result	Overall
Champion et al 2018	High Risk	High Risk	High Risk	Low Risk	Some Concerns	High Risk
Chesak 2015	Some Concerns	High Risk	High Risk	Low Risk	Some Concerns	High Risk
Christopher 2018	Some Concerns	High Risk	Low Risk	Low Risk	Some Concerns	High Risk
Connolly 2020	Low Risk	Some Concerns	Low Risk	Low Risk	Some Concerns	Some Concerns
Crain 2017	Some Concerns	High Risk	High Risk	Low Risk	Some Concerns	High Risk
Cruz-Ferreira 2011	Low Risk	High Risk	Low Risk	Low Risk	Some Concerns	High Risk
Cruz-Ferreira 2015	Low Risk	Some Concerns	Low Risk	Low Risk	Some Concerns	Some Concerns
Danitz 2014	Some Concerns	Some Concerns	High Risk	Low Risk	Some Concerns	High Risk
deVibe 2018	Some Concerns	Low Risk	Low Risk	Low Risk	Some Concerns	Some Concerns
Devillers-Réolon 2022	Some Concerns	High Risk	High Risk	Low Risk	Some Concerns	High Risk
Díaz-Benito 2022	Low Risk	Low Risk	Low Risk	Low Risk	Some Concerns	Some Concerns
Duan 2019	Some Concerns	High Risk	Low Risk	Low Risk	Some Concerns	High Risk
Dvořáková 2017	Some Concerns	Some Concerns	Low Risk	Low Risk	Some Concerns	High Risk
Economides et al 2018	Some Concerns	Some Concerns	Low Risk	Low Risk	Some Concerns	High Risk
Edney 2020	Low Risk	Low Risk	Low Risk	Low Risk	Low Risk	Low Risk

Study Name	1.0 Algorithm Result	2.0 Algorithm result	3.0 Algorithm Result	4.0 Algortithim Result	5.0 Algorithim Result	Overall
Edwards 2019	Some Concerns	High Risk	High Risk	Low Risk	Some Concerns	High Risk
Engel 2019	Some Concerns	Some Concerns	Low Risk	Low Risk	Some Concerns	High Risk
EnriqueRoig 2020	Low Risk	Low Risk	Low Risk	Low Risk	Low Risk	Low Risk
Erogul 2014	Low Risk	Some Concerns	Low Risk	Low Risk	Some Concerns	Some Concerns
Fernández-Portero 2021	Low Risk	Some Concerns	Low Risk	Low Risk	Some Concerns	Some Concerns
Fisher 2004	Some Concerns	Some Concerns	Low Risk	Low Risk	Some Concerns	High Risk
Flook 2013	Some Concerns	High Risk	High Risk	Low Risk	Some Concerns	High Risk
Galante 2016	Low Risk	High Risk	High Risk	Low Risk	Some Concerns	High Risk
Gerodimos 2022	Low Risk	Some Concerns	Low Risk	Low Risk	Some Concerns	Some Concerns
Goldberg et al 2020	Low Risk	Low Risk	Low Risk	Low Risk	Some Concerns	Some Concerns
Green 2006	Some Concerns	Some Concerns	Low Risk	Low Risk	Some Concerns	High Risk
Green 2022	Some Concerns	Some Concerns	Low Risk	Low Risk	Some Concerns	High Risk
Gregoire 2017	Low Risk	Some Concerns	Low Risk	Low Risk	Some Concerns	Some Concerns
Hajatnia 2021	Some Concerns	High Risk	High Risk	Low Risk	Some Concerns	High Risk
Harris 2016	Low Risk	Some Concerns	Low Risk	Low Risk	Some Concerns	Some Concerns
Heintzelman 2019	Some Concerns	Low Risk	Low Risk	Low Risk	Some Concerns	Some Concerns
Hendriks 2020	Low Risk	Some Concerns	Low Risk	Low Risk	Some Concerns	Some Concerns

Study Name	1.0 Algorithm Result	2.0 Algorithm result	3.0 Algorithm Result	4.0 Algortithim Result	5.0 Algorithim Result	Overall
Hilcove 2021	Low Risk	Some Concerns	Low Risk	Low Risk	Some Concerns	Some Concerns
Hirshberg 2018	Low Risk	Some Concerns	Low Risk	Low Risk	Some Concerns	Some Concerns
Hirshberg et al 2022	Low Risk	Low Risk	Low Risk	Low Risk	Low Risk	Low Risk
Ho 2022	Low Risk	Low Risk	Low Risk	Low Risk	Some Concerns	Some Concerns
Hollingsworth 2022	Some Concerns	High Risk	High Risk	Low Risk	Some Concerns	High Risk
Howells et al 2016	Low Risk	High Risk	Low Risk	Low Risk	Low Risk	High Risk
Hu 2017	Low Risk	High Risk	Some Concerns	Some Concerns	Some Concerns	High Risk
Huang 2021	Low Risk	Some Concerns	Low Risk	Some Concerns	Some Concerns	High Risk
Hunsinger 2018	Low Risk	High Risk	Some Concerns	Some Concerns	Some Concerns	High Risk
Hunt 2018	Low Risk	High Risk	Some Concerns	Some Concerns	Some Concerns	High Risk
Hwang 2019	Low Risk	High Risk	Some Concerns	Some Concerns	Some Concerns	High Risk
Ivtzan 2016	Low Risk	High Risk	Some Concerns	Some Concerns	Some Concerns	High Risk
Ivtzan 2018	Low Risk	Low Risk	Low Risk	Some Concerns	Some Concerns	Some Concerns
Janssen 2022	Low Risk	High Risk	Some Concerns	Some Concerns	Some Concerns	High Risk
Janzarik 2022	Low Risk	High Risk	Some Concerns	Some Concerns	Some Concerns	High Risk

Study Name	1.0 Algorithm Result	2.0 Algorithm result	3.0 Algorithm Result	4.0 Algortithim Result	5.0 Algorithim Result	Overall
Jarukasemthawee 2019	Some Concerns	High Risk	Some Concerns	Some Concerns	Some Concerns	High Risk
Jazaieri 2013	Low Risk	High Risk	Some Concerns	Some Concerns	Some Concerns	High Risk
Jazaieri 2014	Low Risk	High Risk	Low Risk	Some Concerns	Some Concerns	High Risk
Jennings 2019	Low Risk	High Risk	Low Risk	Some Concerns	Some Concerns	High Risk
Jones 2019	Some Concerns	High Risk	Low Risk	Some Concerns	Some Concerns	High Risk
Josefsson 2014	Low Risk	High Risk	Some Concerns	Some Concerns	Some Concerns	High Risk
Juul 2021	Low Risk	Low Risk	Low Risk	Some Concerns	Low Risk	Some Concerns
Kadian 2022	Low Risk	Low Risk	Low Risk	Some Concerns	Low Risk	Some Concerns
Kaemmerer 2022	Low Risk	High Risk	Some Concerns	Some Concerns	Some Concerns	High Risk
Kam 2022	Low Risk	Some Concerns	Low Risk	Some Concerns	Some Concerns	High Risk
Kamegaya 2014	Low Risk	High Risk	Some Concerns	Some Concerns	Some Concerns	High Risk
Kang 2022	Low Risk	Some Concerns	Low Risk	Some Concerns	Low Risk	Some Concerns
Karing 2021	Low Risk	Low Risk	Low Risk	Some Concerns	Some Concerns	Some Concerns
Kariyawasam 2022	Some Concerns	Low Risk	Low Risk	Some Concerns	Some Concerns	High Risk

Study Name	1.0 Algorithm Result	2.0 Algorithm result	3.0 Algorithm Result	4.0 Algortithim Result	5.0 Algorithim Result	Overall
Khazaee-Pool 2015	Low Risk	Some Concerns	Low Risk	Some Concerns	Some Concerns	High Risk
Kimura 2010	Low Risk	High Risk	Some Concerns	Some Concerns	Some Concerns	High Risk
Kloos 2022	High Risk	High Risk	Some Concerns	Some Concerns	Low Risk	High Risk
Ko 2018	Low Risk	High Risk	Some Concerns	Some Concerns	Some Concerns	High Risk
Kosugi 2021	Low Risk	Low Risk	Low Risk	Some Concerns	Low Risk	Some Concerns
Krekel 2021	Low Risk	Some Concerns	Low Risk	Some Concerns	Some Concerns	High Risk
Kuhlthau 2020	Low Risk	High Risk	Some Concerns	Some Concerns	Some Concerns	High Risk
Kwon 2015	Some Concerns	Some Concerns	Low Risk	Some Concerns	Some Concerns	High Risk
LeBlanc 2017	Some Concerns	Low Risk	Low Risk	Some Concerns	Some Concerns	High Risk
Lee 2016	Low Risk	High Risk	Some Concerns	Some Concerns	Some Concerns	High Risk
Lee 2019	Low Risk	Low Risk	Low Risk	Some Concerns	Low Risk	Some Concerns
Lee 2021	Low Risk	Low Risk	Low Risk	Some Concerns	Some Concerns	Some Concerns
Li 2001	Low Risk	High Risk	Low Risk	Some Concerns	Some Concerns	High Risk
Li 2015	Low Risk	Low Risk	Low Risk	Some Concerns	Low Risk	Some Concerns

Study Name	1.0 Algorithm Result	2.0 Algorithm result	3.0 Algorithm Result	4.0 Algortithim Result	5.0 Algorithim Result	Overall
Liu 2020	Low Risk	Some Concerns	Low Risk	Some Concerns	Some Concerns	High Risk
Liu 2022 A (LK)	Low Risk	High Risk	Some Concerns	Some Concerns	Some Concerns	High Risk
Liu 2022 B (Exercise)	Low Risk	Low Risk	Low Risk	Low Risk	Low Risk	Low Risk
Loewenthal 2021	Low Risk	High Risk	Some Concerns	Some Concerns	Low Risk	High Risk
Lorenz 2022	Low Risk	High Risk	Low Risk	Some Concerns	Some Concerns	High Risk
Ly 2017	Low Risk	Low Risk	Low Risk	Some Concerns	Some Concerns	Some Concerns
Maatouk 2018	Low Risk	Low Risk	Low Risk	Some Concerns	Low Risk	Some Concerns
Mackenzie 2006	Some Concerns	Some Concerns	Low Risk	Some Concerns	Some Concerns	High Risk
Mak 2015	Low Risk	Low Risk	Low Risk	Some Concerns	Low Risk	Some Concerns
Manotas 2014	Low Risk	High Risk	Some Concerns	Some Concerns	Some Concerns	High Risk
Mascaro 2021	Low Risk	High Risk	Some Concerns	Some Concerns	Low Risk	High Risk
Matos 2022	Some Concerns	High Risk	Some Concerns	Some Concerns	Low Risk	High Risk
Matvienko-Sikar 2017	Low Risk	High Risk	Some Concerns	Some Concerns	Some Concerns	High Risk
Maurer 2020	Low Risk	High Risk	Some Concerns	Some Concerns	Some Concerns	High Risk

Study Name	1.0 Algorithm Result	2.0 Algorithm result	3.0 Algorithm Result	4.0 Algortithim Result	5.0 Algorithim Result	Overall
McConachie 2014	Low Risk	High Risk	Low Risk	Some Concerns	Some Concerns	High Risk
McGonagle 2020	Some Concerns	High Risk	Low Risk	Some Concerns	Some Concerns	High Risk
Miller 2015	Low Risk	High Risk	Some Concerns	Some Concerns	Some Concerns	High Risk
Mirabito 2022	Low Risk	High Risk	Some Concerns	Some Concerns	Some Concerns	High Risk
Montaner 2022	Low Risk	Low Risk	Low Risk	Some Concerns	Some Concerns	Some Concerns
Morledge 2013	Low Risk	High Risk	Some Concerns	Some Concerns	Low Risk	High Risk
Mountain 2017	Low Risk	Low Risk	Low Risk	Some Concerns	Low Risk	Some Concerns
Muller 2016	Low Risk	Low Risk	Low Risk	Some Concerns	Some Concerns	Some Concerns
Murray 2021	Low Risk	Some Concerns	Low Risk	Some Concerns	Some Concerns	High Risk
Myers 2017	Low Risk	Low Risk	Low Risk	Low Risk	Some Concerns	Some Concerns
Nadler 2020	Low Risk	High Risk	Some Concerns	Some Concerns	Some Concerns	High Risk
Nedeljkovic 2012	Some Concerns	High Risk	Some Concerns	Some Concerns	Some Concerns	High Risk
Neff 2013	Low Risk	High Risk	Some Concerns	Some Concerns	Some Concerns	High Risk
Neumeier 2017	Low Risk	High Risk	Some Concerns	Some Concerns	Some Concerns	High Risk
Ng 2016	Low Risk	Some Concerns	Low Risk	Some Concerns	Low Risk	Some Concerns

Study Name	1.0 Algorithm Result	2.0 Algorithm result	3.0 Algorithm Result	4.0 Algortithim Result	5.0 Algorithim Result	Overall
Nguyen 2018	Low Risk	Some Concerns	Low Risk	Some Concerns	Some Concerns	High Risk
Nielsen 2021	Low Risk	High Risk	Some Concerns	Some Concerns	Low Risk	High Risk
Noradechanunt 2017	Low Risk	Low Risk	Low Risk	Low Risk	Some Concerns	Some Concerns
Norman 2010	Low Risk	Some Concerns	Low Risk	Some Concerns	Some Concerns	High Risk
O'Leary 2015	Low Risk	Some Concerns	Low Risk	Some Concerns	Low	High Risk
Oken 2006	Low Risk	Some Concerns	Low Risk	Low Risk	Low	Some Concerns
Oliver, 2018	Low Risk	Low Risk	Low Risk	Some Concerns	Low	Some Concerns
Pacanowski 2020	Low Risk	Low Risk	Low Risk	Some Concerns	Some Concerns	High Risk
Page 2013	Low Risk	Some Concerns	Low Risk	Some Concerns	Some Concerns	High Risk
Pan 2019	Low Risk	Some Concerns	Low Risk	Low Risk	Some Concerns	High Risk
Pang 2019	Low Risk	Low Risk	Low Risk	Some Concerns	Some Concerns	High Risk
Passmore 2022	Low Risk	Some Concerns	Low Risk	Low Risk	High Risk	High Risk
Payne 2020	Low Risk	Some Concerns	Some Concerns	Some Concerns	Some Concerns	High Risk
Perez-Blasco 2016	Low Risk	Some Concerns	Low Risk	Some Concerns	Some Concerns	High Risk
Proyer 2016	Low Risk	Low Risk	Low Risk	Low Risk	Low Risk	Low Risk
Rababah 2022	Low Risk	Some Concerns	Low Risk	Some Concerns	Some Concerns	High Risk

Study Name	1.0 Algorithm Result	2.0 Algorithm result	3.0 Algorithm Result	4.0 Algortithim Result	5.0 Algorithim Result	Overall
Read 2016	Some Concerns	Some Concerns	Low Risk	Some Concerns	Low Risk	High Risk
Recabarren 2019	Low Risk	Low Risk	Low Risk	Low Risk	Low Risk	Low Risk
Rich 2021	Low Risk	Some Concerns	Low Risk	Some Concerns	Low Risk	High Risk
Rodriguez-Jimnez 2022	Low Risk	Some Concerns	Low Risk	Low Risk	Low Risk	Some Concerns
Roeser 2013	Low Risk	Some Concerns	Low Risk	High Risk	High Risk	High Risk
Santos 2022	Low Risk	Some Concerns	Low Risk	Some Concerns	Some Concerns	High Risk
Schulte-Frankenfeld 2022	Low Risk	Some Concerns	Low Risk	Low Risk	Low Risk	Some Concerns
Seear 2013	Low Risk	Some Concerns	Some Concerns	Some Concerns	Some Concerns	High Risk
Senf 2013	Low Risk	Some Concerns	Low Risk	Some Concerns	High Risk	High Risk
Seppälä, 2020	Low Risk	Some Concerns	Low Risk	Low Risk	Low Risk	Some Concerns
Shapiro 2011	Low Risk	Some Concerns	Low Risk	Low Risk	Low Risk	Some Concerns
Sommers-Spijkerman 2018	Low Risk	Low Risk	Low Risk	Low Risk	Low Risk	Low Risk
Sood 2014	Low Risk	Some Concerns	Low Risk	Some Concerns	High Risk	High Risk
Spence 2007	Low Risk	Some Concerns	Low Risk	Some Concerns	Some Concerns	High Risk
Spence 2019	Low Risk	Low Risk	Low Risk	Low Risk	Low Risk	Low Risk
Steinhardt 2008	Low Risk	Some Concerns	Low Risk	Some Concerns	Some Concerns	High Risk
Strauss 2021	Low Risk	Some Concerns	Low Risk	Low Risk	Low Risk	Some Concerns

Study Name	1.0 Algorithm Result	2.0 Algorithm result	3.0 Algorithm Result	4.0 Algortithim Result	5.0 Algorithim Result	Overall
Sturm 2022	Low Risk	Some Concerns	Low Risk	Some Concerns	Low Risk	High Risk
Taylor 2014	Low Risk	Low Risk	Low Risk	Some Concerns	Low Risk	Some Concerns
Taylor 2022	Low Risk	Some Concerns	Low Risk	Some Concerns	Low Risk	Some Concerns
Terblanche 2022	Low Risk	Some Concerns	Low Risk	High Risk	Low Risk	High Risk
Timlin 2017	Low Risk	Some Concerns	Low Risk	Some Concerns	Low Risk	High Risk
Trombka 2021	Low Risk	Some Concerns	Low Risk	Low Risk	Low Risk	Some Concerns
Tsang 2021	Low Risk	Some Concerns	Low Risk	Low Risk	Low Risk	Some Concerns
Van Roie 2017	Low Risk	Some Concerns	Low Risk	Some Concerns	Low Risk	High Risk
vanDijk 2017	Low Risk	Low Risk	Low Risk	Some Concerns	Low Risk	Some Concerns
vanEmmerik 2018	Low Risk	Low Risk	Low Risk	Low Risk	Low Risk	Low Risk
Verweij 2018	Low Risk	Some Concerns	Low Risk	Some Concerns	Low Risk	High Risk
Viskovich 2020	Low Risk	Low Risk	Low Risk	Low Risk	Low Risk	Low Risk
Wadhen 2021	Low Risk	Some Concerns	Low Risk	Some Concerns	Low Risk	High Risk
Waelde 2017	Low Risk	Low Risk	Low Risk	Some Concerns	Some Concerns	High Risk
Wang 2020	Low Risk	Some Concerns	Low Risk	Some Concerns	Low Risk	High Risk
Welford 2022	Low Risk	Low Risk	Low Risk	Low Risk	Low Risk	Low Risk
Weytens 2014	Low Risk	Some Concerns	Low Risk	Some Concerns	Some Concerns	High Risk

Study Name	1.0 Algorithm Result	2.0 Algorithm result	3.0 Algorithm Result	4.0 Algortithim Result	5.0 Algorithim Result	Overall
Wilke 2022	Low Risk	Some Concerns	Low Risk	Low Risk	Low Risk	Some Concerns
Wingert 2022	Low Risk	Some Concerns	Low Risk	Some Concerns	Low Risk	Some Concerns
Xiong 2022	High Risk	Some Concerns	Low Risk	Some Concerns	Low Risk	High Risk
Xu 2021	Low Risk	Low Risk	Low Risk	Low Risk	Low Risk	Low Risk
Yang 2018	Low Risk	High Risk	Low Risk	Low Risk	Low Risk	High Risk
Yıldırım	Low Risk	Low Risk	Low Risk	Some Concerns	Some Concerns	High Risk
Zeng 2019	Low Risk	Low Risk	Low Risk	Some Concerns	Some Concerns	High Risk
Zhang 2019	Low Risk	Some Concerns	Low Risk	Some Concerns	Low Risk	High Risk
Zheng 2022	Low Risk	Low Risk	Low Risk	Some Concerns	Low Risk	Some Concerns
Zilcha-Mano 2016	Low Risk	Some Concerns	Low Risk	Some Concerns	Low Risk	High Risk

2. Results

2.1. Original node labels and brief descriptions: those in BOLD were excluded from final analysis or merged with another node following transitivity and consistency checks.

Node Label	Intervention Brief Description
C	No Intervention Control - includes passive control (e.g., sit still), no intervention and wait list and TAU
MIND	Mindfulness-based approaches
EX	Exercise
COMB	Multi-Component Psychological intervention (e.g. combined CBT, PPI and Mindfulness). Clear psychological paradigms combined into one.
PPI	Single component PPI e.g. Three good things exercise, character strengths, best possible self or goal setting
ACT	Acceptance and commitment therapy
NAT	Nature Interventions
EXPSY	Physical movement with a psychological intervention (excludes yoga)
COMPAS	Compassion focused therapy
YOGA	Yoga
MPPI	Multi-Component PPI
**MEMS**	**Early Memories / Reminiscence**
**PE**	**Psychoeducation**
**ACTIVE**	**Active Control – includes listening to audiobook, writing down neutral events, daily activities, self-monitoring**
**GOAL**	**Goal Setting Interventions**

**STRENGTH**	**Character Strengths**
**SOCIAL**	**Social Groups e.g., discussions, peer support, sharing stressors**
**HE**	**Physical health or lifestyle Education**
**CBT**	**Cognitive Behavioural Therapy**
**GRAT**	**Gratitude**
**BPS**	**Best Possible Self**
**EXNAT**	**Exercise in nature**
**WRITE**	**Expressive Writing**

*Nodes highlighted in bold were adapted, merged, or removed (additional information in Supplementary Information 2.2.3

2.2. Node adaptions

2.2.1. Initial assessment of transitivity prior to node exclusion (box plot distribution of potential effect modifier: age)

SFigure 2
Boxplot of Sample Age Distributions by Comparison

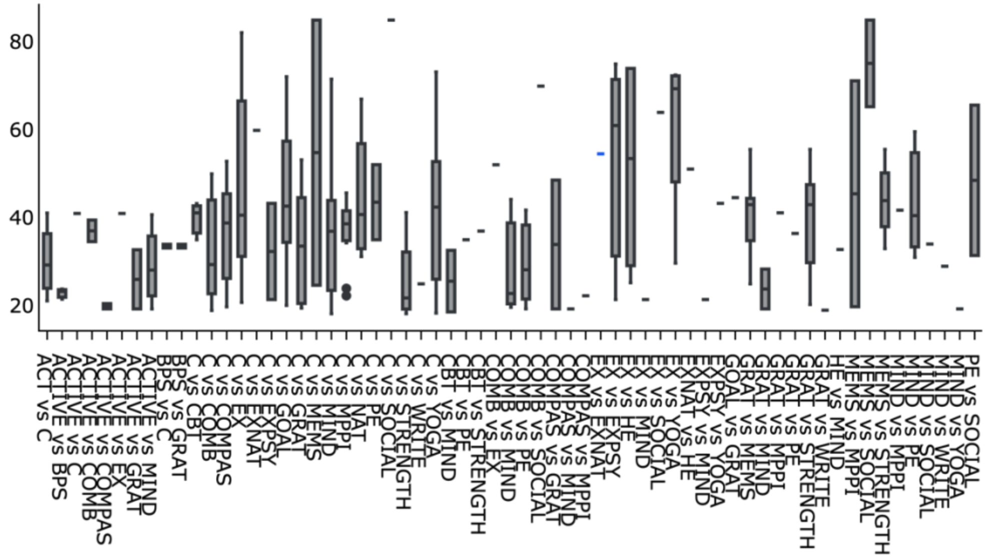

Note: Sample sizes vary across intervention pairs; boxplots show median and range within each direct comparison.

2.2.2. Initial assessment of local inconsistency prior to node exclusion

Comparison	direct	indirect	p-value
ACTIVE vs BPS	0.9035	0.7412	0.6036
ACTIVE vs C	0.9299	1.3136	0.3457
ACTIVE vs COMB	0.6904	0.8857	0.4019
ACTIVE vs COMPAS	0.9048	0.7866	0.6684
ACTIVE vs EX	0.804	0.8674	0.8397
ACTIVE vs GRAT	0.8596	0.9145	0.8389

ACTIVE vs MIND	0.9366	0.7726	0.451
BPS vs C	1.8254	1.3295	0.4227
BPS vs GRAT	1.0764	1.0707	0.9897
C vs CBT	0.9645	0.6066	0.1017
C vs COMB	0.6687	0.6248	0.7032
C vs COMPAS	0.6283	0.721	0.5401
C vs EX	0.6882	0.6558	0.7871
C vs EXNAT	0.5073	0.55	0.8893
C vs EXPSY	0.4153	0.5112	0.6338
C vs GOAL	0.6499	1.4103	0.3032
C vs GRAT	0.7083	0.7197	0.9296
C vs MEMS	0.4954	0.9112	0.0978
C vs MIND	0.6518	0.6829	0.719
C vs MPPI	0.6202	0.3658	0.0609
C vs PE	0.9806	0.7337	0.3227
C vs SOCIAL	0.5654	1.275	0.0566
C vs STRENGTH	0.6887	0.7004	0.9488
C vs WRITE	0.8281	0.7536	0.8527
C vs YOGA	0.8778	0.5679	0.123
COMB vs EX	0.8611	1.0508	0.6108
COMB vs MIND	1.0917	0.9837	0.6737
COMB vs PE	1.112	1.2203	0.7257
COMB vs SOCIAL	1.172	1.7471	0.4396
COMPAS vs EX	1.0691	1.0501	0.9627
COMPAS vs GRAT	0.8407	1.1864	0.2504
COMPAS vs MIND	1.0735	1.0111	0.884
COMPAS vs MPPI	0.9218	0.8764	0.9056
EX vs EXNAT	0.3477	1.029	0.0827
EX vs EXPSY	0.7242	0.6295	0.7638
EX vs HE	1.2534	0.8036	0.2345
EX vs MIND	0.8762	0.9686	0.8243
EX vs SOCIAL	1.532	1.6143	0.9094
EX vs YOGA	0.9228	1.3254	0.1856
EXNAT vs HE	0.9518	2.2905	0.1037
EXPSY vs MIND	1.1796	1.4485	0.6836

EXPSY vs YOGA	1.9144	1.6634	0.816
GOAL vs GRAT	0.9354	1.0982	0.7132
GRAT vs MEMS	1.0826	1.4915	0.2196
GRAT vs MIND	0.8568	0.9267	0.8115
GRAT vs MPPI	1.1941	0.7519	0.2273
GRAT vs PE	1.0061	1.0982	0.8556
GRAT vs STRENGTH	0.9677	0.9822	0.9555
GRAT vs WRITE	1.4641	0.912	0.3409
HE vs MIND	0.8863	0.8578	0.9414
MEMS vs MPPI	0.2932	0.877	0.0019
MEMS vs SOCIAL	1.1998	1.3296	0.8179
MEMS vs STRENGTH	0.8738	0.7727	0.6787
MIND vs CBT	0.85	1.3873	0.1064
MIND vs MPPI	0.974	0.8572	0.7291
MIND vs PE	1.0899	1.2461	0.5816
MIND vs SOCIAL	17.6293	1.2886	0
MIND vs WRITE	0.8253	1.4418	0.2592
MIND vs YOGA	1.0371	1.2614	0.5939
PE vs CBT	1.1214	0.9584	0.7056
PE vs SOCIAL	1.2018	1.5616	0.4699
STRENGTH vs CBT	1.115	1.1093	0.9906

2.2.3. Rationale for node adaptions made based on transitivity and consistency assessments.

SOCIAL

Multiple comparisons with the node SOCIAL (social identity building interventions) consistently had an average age of participants higher than other comparisons. For example, the comparison MEMS v SOCIAL had a median age of 75.5yrs old (see Supplementary Information 2.1.1). In addition, during SIDE splitting checks, two comparisons containing SOCIAL (MIND v SOCIAL, C v SOCIAL) were found to be inconsistent (direct and indirect estimates were statistically significantly different). Following a discussion concerning

the clinical and statistical heterogeneity of the interventions classed as SOCIAL, the decision was made to exclude the intervention node SOCIAL from the NMA.

GRAT

This node originally contained the classic gratitude intervention ‘three good things’ in addition to savouring interventions and one ‘gratitude visit’ intervention. Upon discussion, it was decided that these intervention types were too distinct to be classed as one node. Since there were not enough arms to create a new node, gratitude visit (n = 1) and savouring intervention arms (n = 2) were removed from NMA.

MEMS

This node originally contained both reminiscence discission groups and early memories interventions. From clinical discussion, it was decided that discussion groups should be re-categorised as SOCIAL (a node which was later removed) and early memories intervention arms should be characterised in the ACTIVE control node.

MPPI

MPPI was re-defined to include interventions which explicitly contained positive psychology interventions such as acts of kindness, gratitude, mindfulness, resilience, goal setting, strengths building. Treatment arms which described themselves as positive psychology but whose content had been significantly adapted to a niche client group were removed (e.g. positive psychology for couples therapy or tailored to manage older aging). The aim of this re-definition was to reduce the heterogeneity of interventions defined as MPPI and ensure they made clinical sense to categorise as one node. 3 arms in total were removed.

CBT

Arms defined as CBT were checked to ensure they fitted a description of CBT. We found only n=2 arms truly fitted the definition of CBT; the others were more broadly related to challenging thought patterns but did not explicitly fit the CBT model. In addition, during

discussion amongst the research group, concerns were raised that CBT is not a wellbeing focused psychological intervention, given that its emphasis is on reducing ill being (via eliminating unhelpful thoughts and behaviours) as opposed to building positive wellbeing. The decision was therefore made to remove the studies within the node CBT.

EXNAT

Three eligible studies were identified for this node, however each of which was conceptually distinct, limiting the potential for meaningful synthesis. One involved a structured park prescription programme that included lifestyle counselling (Müller-Riemenschneider et al., 2020), another examined immersive multi-day outdoor camping activities on a remote island (Lai et al., 2022), and the third tested the impact of viewing a nature video while cycling indoors (Calogiuri et al., 2021). These interventions varied significantly in both psychological focus and intensity of nature exposure, precluding the formation of a coherent and interpretable EXNAT node within the NMA. As such, this category was excluded from the final model.

PPI

Following discussion, the decision was made to merge the single PPIs to one node, given that their effect size estimates were similar, to increase the number of arms in a node and increase the confidence of the estimate. Therefore GOAL, GRAT, STRENGTH and BPS were all changed to PPI. Where one study compared two PPIs versus a control in multiple arms, the PPI which was most like the other interventions in the node were chosen for inclusion.

PE/HE

Psychoeducation and health education were merged to create one node called ED to increase the number of studies in the node.

ACTIVE/WRITE

It was decided that it did not make clinical sense to cluster the interventions in the active control condition together as they were heterogeneous in practice e.g. listening to an audiobook versus writing about your typical day, versus reflecting on memories, verses

reflective journaling. When the active controls were further split into separate nodes, the n of arms became very small and had large confidence intervals. It was decided eventually to remove active controls from the overall NMA.

2.3. Characteristics of Included Studies Across the Different Comparisons

Comparison	Setting: Community (%)	Setting: Online (%)	Setting: University (%)	Setting: Workplace (%)	Intensity: Brief (%)	Intensity: Short (%)	Intensity: Medium (%)	Intensity: Long (%)	Delivery: In-person (%)	Delivery: Online platform (%)	Delivery: Instructions (%)	Delivery: Live video (%)	Format: Group (%)	Format: Individual (%)	Western (%)
COMB-MIND	0%	0%	50%	50%	0%	0%	100%	0%	100%	0%	0%	0%	100%	0%	100%
COMPAS-PPI	0%	0%	100%	0%	100%	0%	0%	0%	100%	0%	0%	0%	0%	100%	100%
C-ACT	0%	0%	60%	40%	20%	40%	20%	20%	80%	20%	0%	0%	80%	20%	80%
C-COMB	15%	15%	50%	20%	0%	20%	75%	6.7%	57.9%	36.8%	5.3%	0%	65%	35%	85%
C-COMPAS	26.7%	13.3%	26.7%	33.3%	0%	20%	60%	25%	78.6%	14.3%	0%	7.1%	71.4%	28.6%	64.3%
C-ED	0%	0%	0%	100%	0%	0%	100%	0%	100%	0%	0%	0%	100%	0%	100%
C-EX	45.5%	9.1%	36.4%	13.6%	5%	12.5%	16.7%	62.5%	81%	9.5%	4.8%	4.8%	68.2%	31.8%	77.3%
C-EXPSY	0%	0%	100%	0%	100%	0%	0%	0%	100%	0%	0%	0%	0%	100%	100%
C-MIND	5.4%	18%	45%	45%	11.7%	25%	63.3%	6.7%	66.2%	29.2%	3.1%	0%	68.8%	31.2%	82.8%
C-MPPI	25%	0%	37.5%	37.5%	12.5%	12.5%	62.5%	12.5%	75%	0%	0%	25%	62.5%	37.5%	87.5%
C-NAT	50%	0%	50%	0%	25%	50%	0%	25%	75%	0%	0%	25%	50%	50%	50%
C-PPI	33.3%	22.2%	27.8%	11.1%	30%	20%	20%	20%	47.1%	17.6%	0%	35.3%	33.3%	66.7%	72.2%
C-YOGA	27.3%	0%	27.3%	45.5%	0%	16.7%	41.7%	41.7%	91.7%	0%	8.3%	0%	100%	0%	100%
ED-COMB	0%	0%	33.3%	66.7%	0%	100%	0%	0%	66.7%	33.3%	0%	0%	66.7%	33.3%	66.7%
ED-EX	60%	0%	40%	0%	0%	0%	0%	100%	100%	0%	0%	0%	80%	20%	60%
ED-MIND	20%	20%	0%	60%	40%	0%	40%	20%	60%	40%	0%	0%	60%	40%	75%
ED-PPI	0%	0%	100%	0%	0%	100%	0%	0%	0%	0%	0%	100%	0%	100%	100%
EX-COMB	100%	0%	0%	0%	0%	0%	0%	100%	100%	0%	0%	0%	100%	0%	100%
EX-COMPAS	0%	100%	0%	0%	0%	100%	0%	0%	0%	100%	0%	0%	0%	100%	100%
EX-EXPSY	66.7%	0%	33.3%	0%	33.3%	0%	33.3%	33.3%	66.7%	33.3%	0%	0%	0%	100%	66.7%
EX-MIND	0%	0%	100%	0%	100%	0%	0%	0%	100%	0%	0%	0%	0%	100%	100%
EX-YOGA	100%	0%	0%	0%	0%	0%	0%	100%	100%	0%	0%	0%	100%	0%	100%
MIND-COMPAS	0%	0%	100%	0%	100%	0%	0%	0%	100%	0%	0%	0%	0%	100%	100%
MIND-C	0%	0%	100%	0%	0%	0%	0%	0%	0%	0%	0%	0%	0%	0%	100%

Comparison	Setting: Community (%)	Setting: Online (%)	Setting: University (%)	Setting: Workplace (%)	Intensity: Brief (%)	Intensity: Short (%)	Intensity: Medium (%)	Intensity: Long (%)	Delivery: In-person (%)	Delivery: Online platform (%)	Delivery: Instructions (%)	Delivery: Live video (%)	Format: Group (%)	Format: Individual (%)	Western (%)
MIND-EXPSY	0%	0%	100%	0%	100%	0%	0%	0%	100%	0%	0%	0%	0%	100%	100%
MIND-PPI	0%	0%	100%	0%	100%	0%	0%	0%	100%	0%	0%	0%	0%	100%	100%
MIND-YOGA	0%	0%	100%	0%	0%	50%	50%	0%	100%	0%	0%	0%	100%	0%	100%
MPPI-COMPAS	0%	0%	100%	0%	0%	0%	100%	0%	0%	0%	0%	0%	0%	0%	100%
MPPI-PPI	100%	0%	0%	0%	100%	0%	0%	0%	0%	0%	0%	100%	0%	100%	100%
PPI-COMPAS	100%	0%	0%	0%	0%	0%	100%	0%	0%	0%	0%	100%	0%	100%	100%
PPI-MIND	0%	100%	0%	0%	0%	100%	0%	0%	0%	100%	0%	0%	0%	100%	100%
YOGA-EX	50%	0%	50%	0%	0%	0%	0%	100%	100%	0%	0%	0%	100%	0%	100%

2.4. Final NMA Model results including final SIDE assessment of local consistency (coherence)

Comparison	N of studies direct evidence	Network meta-analysis	Direct estimate	Indirect estimate	Difference	Incoherence (p value)
ACT:COMB	0	-0.0061	.	-0.0061	.	.
ACT:COMPAS	0	-0.0542	.	-0.0542	.	.
ACT:C	5	0.3920	0.3920	.	.	.
ACT:ED	0	0.1348	.	0.1348	.	.
ACT:EX	0	-0.0263	.	-0.0263	.	.
ACT:EXPSY	0	-0.3430	.	-0.3430	.	.
ACT:MIND	0	-0.0518	.	-0.0518	.	.
ACT:MPPI	0	0.0821	.	0.0821	.	.
ACT:NAT	0	0.3447	.	0.3447	.	.
ACT:PPI	0	-0.0146	.	-0.0146	.	.
ACT:YOGA	0	-0.1025	.	-0.1025	.	.
COMB:COMPAS	0	-0.0481	.	-0.0481	.	.
COMB:C	21	0.3981	0.4031	0.3733	0.0298	0.8935
COMB:ED	3	0.1409	0.1107	0.1549	-0.0442	0.8693
COMB:EX	1	-0.0202	-0.1496	-0.0090	-0.1405	0.7280
COMB:EXPSY	0	-0.3369	.	-0.3369	.	.
COMB:MIND	2	-0.0457	0.0254	-0.0529	0.0783	0.8101
COMB:MPPI	0	0.0882	.	0.0882	.	.
COMB:NAT	0	0.3509	.	0.3509	.	.
COMB:PPI	0	-0.0084	.	-0.0084	.	.
COMB:YOGA	0	-0.0964	.	-0.0964	.	.
COMPAS:C	15	0.4462	0.4661	0.3475	0.1186	0.6451
COMPAS:ED	0	0.1890	.	0.1890	.	.
COMPAS:EX	1	0.0279	0.0668	0.0234	0.0434	0.9132
COMPAS:EXPSY	0	-0.2888	.	-0.2888	.	.
COMPAS:MIND	1	0.0024	0.0702	-0.0026	0.0728	0.8636
COMPAS:MPPI	1	0.1363	-0.0815	0.1764	-0.2578	0.5660

COMPAS:NAT	0	0.3989	.	0.3989	.	.
COMPAS:PPI	2	0.0397	-0.1727	0.0959	-0.2686	0.3851
COMPAS:YOGA	0	-0.0483	.	-0.0483	.	.
ED:C	1	0.2572	0.1085	0.2703	-0.1618	0.6902
EX:C	23	0.4183	0.3780	0.5394	-0.1613	0.3735
EXPSY:C	1	0.7350	0.8257	0.7018	0.1238	0.8166
MIND:C	65	0.4438	0.4463	0.4178	0.0285	0.8697
MPPI:C	8	0.3099	0.3111	0.2947	0.0163	0.9752
NAT:C	4	0.0472	0.0472	.	.	.
PPI:C	18	0.4065	0.4048	0.4171	-0.0123	0.9618
YOGA:C	12	0.4945	0.4410	0.7745	-0.3335	0.3074
ED:EX	5	-0.1611	-0.2717	-0.0829	-0.1888	0.4329
ED:EXPSY	0	-0.4778	.	-0.4778	.	.
ED:MIND	5	-0.1866	-0.0922	-0.2500	0.1578	0.4934
ED:MPPI	0	-0.0527	.	-0.0527	.	.
ED:NAT	0	0.2099	.	0.2099	.	.
ED:PPI	1	-0.1493	-0.0061	-0.1631	0.1570	0.7488
ED:YOGA	0	-0.2373	.	-0.2373	.	.
EX:EXPSY	3	-0.3167	-0.3197	-0.2943	-0.0254	0.9715
EX:MIND	1	-0.0255	-0.1321	-0.0211	-0.1110	0.8101
EX:MPPI	0	0.1084	.	0.1084	.	.
EX:NAT	0	0.3710	.	0.3710	.	.
EX:PPI	0	0.0118	.	0.0118	.	.
EX:YOGA	3	-0.0762	-0.1698	-0.0411	-0.1287	0.6742
EXPSY:MIND	1	0.2912	0.1651	0.3400	-0.1749	0.7430
EXPSY:MPPI	0	0.4251	.	0.4251	.	.
EXPSY:NAT	0	0.6877	.	0.6877	.	.
EXPSY:PPI	0	0.3285	.	0.3285	.	.
EXPSY:YOGA	0	0.2405	.	0.2405	.	.
MIND:MPPI	0	0.1339	.	0.1339	.	.
MIND:NAT	0	0.3965	.	0.3965	.	.
MIND:PPI	2	0.0373	0.1590	0.0241	0.1349	0.6889

MIND:YOGA	2	-0.0507	0.0355	-0.0637	0.0992	0.7930
MPPI:NAT	0	0.2626	.	0.2626	.	.
MPPI:PPI	1	-0.0966	-0.1774	-0.0785	-0.0988	0.8093
MPPI:YOGA	0	-0.1846	.	-0.1846	.	.
NAT:PPI	0	-0.3593	.	-0.3593	.	.
NAT:YOGA	0	-0.4472	.	-0.4472	.	.
PPI:YOGA	0	-0.0880	.	-0.0880	.	.

3. Moderator Analyses using Meta-regressions

To explore potential sources of heterogeneity in wellbeing outcomes, we conducted a series of univariable mixed-effects meta-regression models using the *metafor* package in R. Standardised mean differences and standard errors from pairwise comparisons of interventions versus control were used. Between-study variance was estimated using REML, and statistical significance was evaluated with Knapp–Hartung adjustments.

The following moderators were examined:

- Intervention intensity (Brief <2 weeks, Short 2–4 weeks, Medium 5–8 weeks, Long >8 weeks)
- Delivery mode (in-person, live online, platform, instruction)
- Format (individual vs. group)
- Setting (community, university, workplace, other)
- Country (Western vs. non-Western)
- Participant age (continuous mean age per trial)

Results

- Intervention intensity: Intensity significantly moderated wellbeing outcomes ($F(3,167) = 4.38$, $p = .0054$). Medium-length interventions (5–8 weeks) showed stronger effects than Short interventions (estimate = –0.354, 95% CI [–0.560, –0.148], $p < .001$). Other contrasts were non-significant, though the pattern suggested Medium interventions tended to outperform both Brief and Long formats.
- Delivery mode: No significant moderation was detected ($F(3,163) = 0.47$, $p = .70$).
- Format: Group vs. individual delivery showed no significant differences ($F(1,167) = 0.65$, $p = .42$).
- Setting: No significant moderation by study setting was observed ($F(3,166) = 1.14$, $p = .33$).
- Country: Western vs. non-Western samples showed no significant difference ($F(1,168) = 0.98$, $p = .32$).
- Age: Mean participant age did not significantly moderate outcomes (estimate = 0.0015, 95% CI [–0.0045, 0.0074], $p = .62$).

3.1. Table summary of meta-regression results

Moderator	Reference Category	Significant Contrasts	τ^2	I^2	R^2	Overall F-test (p)
Intervention intensity (length2)	Short	Medium > Short (p = .0009)	0.171	81%	8.7%	F(3,167) = 4.38, p = .005
Delivery mode (delivery2)	In-person	None	0.198	83%	0%	F(3,163) = 0.47, p = .70
Format (format2)	Individual	None	0.192	83%	0%	F(1,167) = 0.65, p = .42
Setting (setting)	University	None	0.188	82%	0.9%	F(3,166) = 1.14, p = .33
Region (western)	Western	None	0.163	80%	0%	F(1,168) = 0.98, p = .32
Mean age (continuous)	–	None (slope = 0.0015, p = .62)	0.205	83%	0%	F(1,158) = 0.25, p = .62

Interpretation

Among the tested moderators, only intervention intensity significantly explained variability in outcomes. No consistent evidence was found that delivery mode, group/individual format, setting, cultural context, or participant age moderated effects. Across models, residual heterogeneity remained high (I^2 = 80–83%), reflecting substantial unexplained between-study variability. Moderator R^2 values indicated that intensity accounted for approximately 9% of heterogeneity, whereas other moderators explained <1%. This suggests that while intervention length is a meaningful factor, additional unmeasured variables likely contribute to outcome differences across trials.

3.2. Intervention x Moderator Interactions

To assess whether moderator effects might differ by intervention type, we conducted exploratory interaction meta-regressions. Because of data sparsity in many intervention x moderator cross-classifications (e.g., fewer than 3 studies in several cells) we did a systematic feasibility check using tabulations and then restricted analyses to the four intervention types most widely represented across moderators: Mindfulness, Exercise, Combined Psychological Interventions, and Single PPIs. This ensured that each intervention x moderator cell included at least three studies.

We tested interactions between intervention type and four key moderators:

- Delivery mode (in-person, instruction-based, online platform),
- Format (group vs. individual delivery),
- Study setting (Western vs. non-Western countries).
- Intensity (Brief, Short, Medium v Long length interventions)

All models were fit as mixed-effects meta-regressions (REML estimation, Knapp–Hartung adjustments) using rma() in the metafor package.

3.2.1. Table of Intervention X Moderator Meta-Regression Results

Moderator	k (studies)	I^2 (%)	τ^2	Test of Moderators	R^2 (%)	Conclusion
Delivery × Treatment	114	84.7	0.247	$F(7,106) = 0.15$, $p = .99$	0.0	No significant interaction
Format × Treatment	124	84.8	0.237	$F(7,116) = 0.15$, $p = .99$	0.0	No significant interaction
Western × Treatment	125	81.9	0.195	$F(7,117) = 0.16$, $p = .99$	0.0	No significant interaction
Intensity x Treatment	123	82%		$F(12, 110) = 2.27$, $p = 0.013$	11.9	Only Exercise × Medium vs Short was significant (estimate = –1.18, *p* = .03)

Sparse data for other intervention types and moderator categories limited our ability to test interactions. These exploratory analyses

indicate that, within the limits of available data, intervention effectiveness did not systematically differ by delivery mode, group/individual format, or study setting (Western vs. non-Western).

For intensity, the overall model was statistically significant ($F(12, 110) = 2.27$, $p = .013$), accounting for 12% of between-study heterogeneity ($R^2 = 11.9\%$). However, most interaction terms were non-significant with wide confidence intervals. The only significant effect suggested that medium-length Exercise interventions were less effective than short Exercise interventions (estimate = –1.18, 95% CI [–2.25, –0.12], $p = .03$). Given multiple testing and sparsity of data in several cells, this result should be interpreted very cautiously and considered exploratory only.

4. Sensitivity Analyses

For moderator or sensitivity analyses with multiple categorical levels we used meta-regression because it provides a unified framework for testing differences across groups. By contrast, for specific sensitivity analyses (e.g., low risk of bias category, SWB outcome measure), we also re-ran full NMA models because results were not sparse (sufficient studies per group), and we wanted to test robustness of the network structure under different assumptions. This dual approach let us apply meta-regression where it was most informative (moderators with multiple levels) while reserving full alternative NMA runs for the three sensitivity checks where they were both computationally feasible and methodologically justified.

4.1. Sensitivity Analyses using Meta-Regression

We conducted sensitivity analyses using mixed-effects meta-regressions (active interventions vs. control), with REML estimation and Knapp–Hartung adjustments.

Outcome measure type

The wellbeing outcome assessed (subjective wellbeing, resilience, mindfulness, positive affect) did not significantly moderate intervention effects ($F(3,169) = 1.08$, $p = .36$). Residual heterogeneity remained high ($I^2 = 82.4\%$, $\tau^2 = 0.18$), indicating that differences in outcome type do not explain between-study variability.

Control group type
No difference was observed between trials using waitlist controls and those using no-intervention controls ($F(1,170) = 0.00$, $p = .98$; $I^2 = 82.6\%$, $\tau^2 = 0.19$). Thus, control condition does not appear to account for heterogeneity in effect sizes.

Risk of bias category
Risk of bias ratings (low, medium, high) were not a significant moderator ($F(2,170) = 1.78$, $p = .17$). Although effect sizes tended to be somewhat larger in medium-risk studies, these differences were not statistically reliable. Residual heterogeneity remained high ($I^2 = 82.1\%$, $\tau^2 = 0.19$).

4.1.1. Table of results: sensitivity analyses using mixed-effects meta-regression

Moderator	Levels compared	Test of moderators (F)	p-value	τ^2	I^2 (%)	Interpretation
Outcome measure type	SWB, Resilience, Mindfulness, Positive affect	F(3,169) = 1.08	.36	0.18	82.4	No evidence outcome type moderates effects
Control group type	Waitlist vs No-intervention	F(1,170) = 0.00	.98	0.19	82.6	No difference between control types
Risk of bias category	Low, Medium, High	F(2,170) = 1.78	.17	0.19	82.1	No significant moderation by risk of bias

Summary
Across sensitivity analyses, outcome measure type, control condition, and risk of bias did not significantly moderate effects. These findings reinforce the robustness of the primary results, while highlighting that much of the between-study variability remains unexplained.

4.2 Sensitivity analysis using NMA models

4.2.1 Network meta-analysis model results when studies containing high risk of bias are excluded.

The network was relatively connected, only one subnetwork was present, however 4/12 interventions only had one direct comparison (not strongly attached). The model contained 73 studies, 12 treatments and 81 pairwise comparisons. The design-by-treatment interaction model suggested global inconsistency in the network ($tau^2 = 0.1144$; tau = 0.3383; $I^2 = 78\%$ [72.4%; 82.5%], $p < 0.001$). However, under the assumption of a full design-by-treatment interaction random effects model, Q decreased considerably, and between-design inconsistency was no longer significant (Q = 14.10, $p = 0.228$).

In the low-medium risk of bias model, four out of five of the top ranked treatments remained the same as the main model. The one modification was that ACT now ranked higher in the top five. In addition, single positive psychology interventions (PPIs) fell significantly in ranking when high risk of bias studies were removed, however remained significantly more effective than control condition ($p = 0.049$). Multi-component PPIs had a larger confidence interval and was no longer significant compared to controls ($p = 0.186$).

SFigure 3
Forest plot showing the standardized mean differences (SMD) for each intervention versus control after removing studies rated high risk of bias.

Treatment	Comparisons	SMD	95%-CI	P-score
Exercise & Psychological	0	1.36	[0.57; 2.15]	0.98
Yoga	4	0.68	[0.29; 1.07]	0.77
Exercise	11	0.51	[0.29; 0.74]	0.60
Mindfulness	27	0.49	[0.35; 0.64]	0.57
Acceptance & Commitment	3	0.51	[0.09; 0.92]	0.57
Compassion	2	0.48	[0.04; 0.92]	0.54
Combined Psych	12	0.44	[0.23; 0.65]	0.48
Multi-Component PPI	2	0.36	[-0.17; 0.89]	0.40
Educational	1	0.38	[0.07; 0.70]	0.40
Single PPI	3	0.37	[0.00; 0.74]	0.39
Nature	2	0.23	[-0.34; 0.81]	0.28
Control	0	0.00		0.03

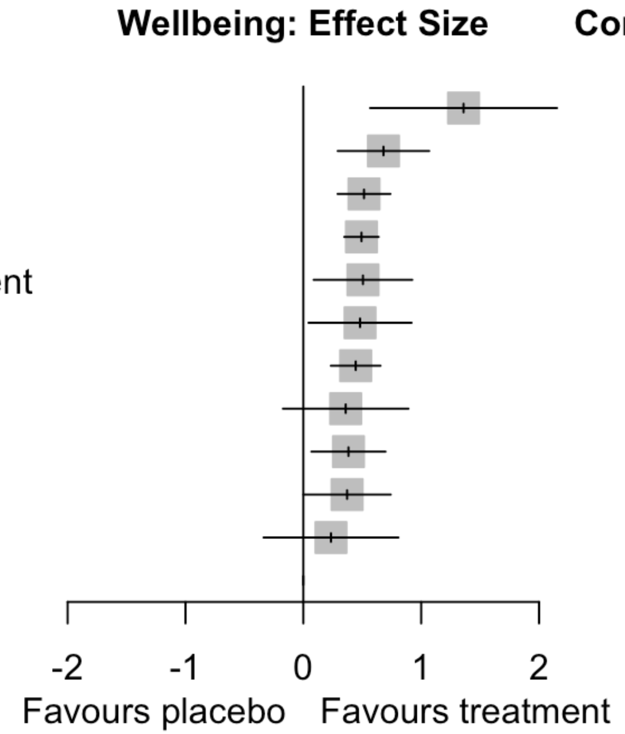

Note: Interventions ordered by P-score ranking. The “Comparisons” column indicates the number of studies with direct comparisons between each intervention and control. Estimates for interventions with k=0 are derived entirely from indirect evidence within the network.

4.2.2 Network meta-analysis model results only using SWB as outcome measure

Network was well connected and comparable to main model. It contained 117 studies, 133 pairwise comparisons and 12 treatments. The design-by-treatment interaction model suggested global inconsistency in the network ($tau^2 = 0.0930$; tau = 0.3050; $I^2 = 72.9\%$

[67.5%; 77.5%], $p < 0.001$). Under the assumption of a full design-by-treatment interaction random effects model, Q was no longer significant (Q = 8.76, $p = 0.978$).

Model results were comparable with the main NMA. Again, four out of five of the top ranked treatments remained the same, however ACT increased in effect size (0.39 to 0.50) and rank from eighth to second. Single PPIs and multi-component PPIs ranked more comparably when SWB was used at main outcome measure.

SFigure 4

Sensitivity Analysis: Forest Plot including only Subjective Wellbeing as the outcome measure

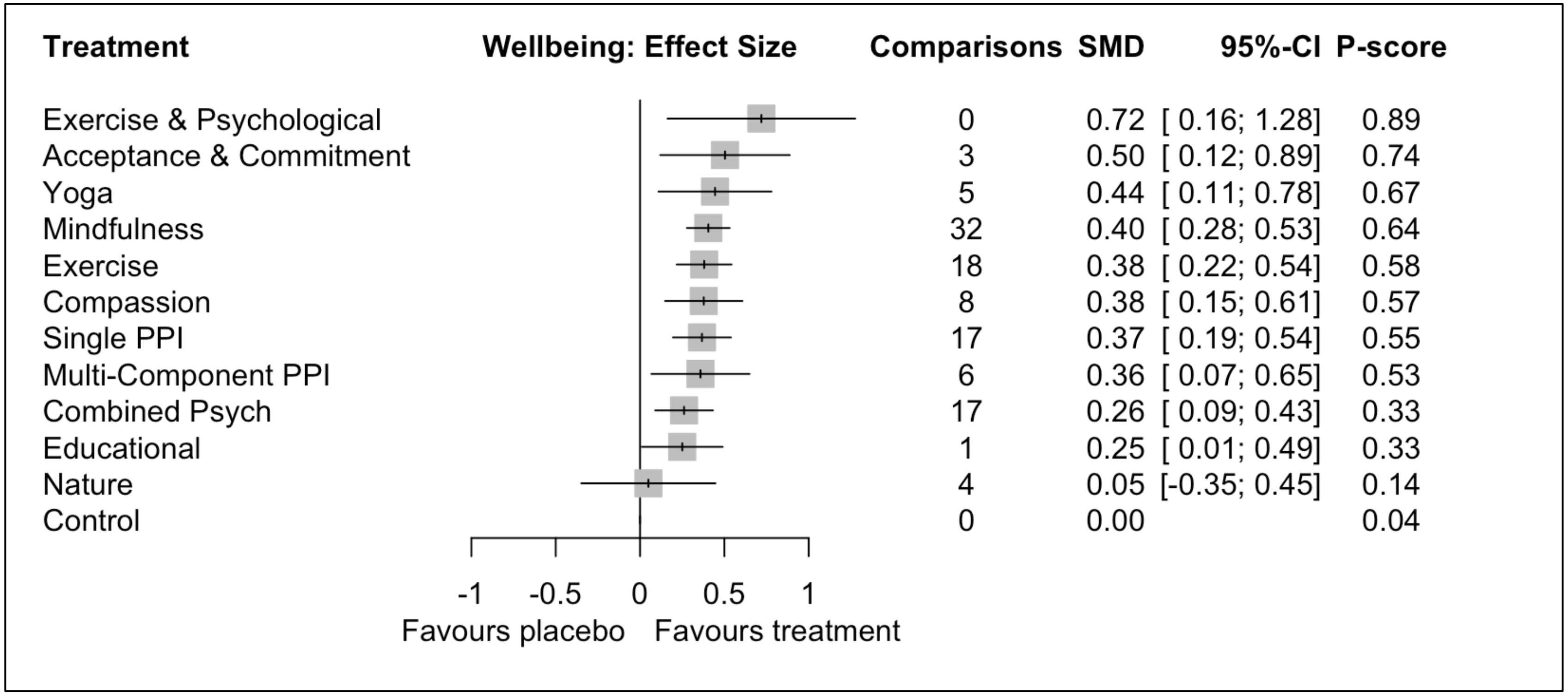

Note: Interventions ordered by P-score ranking. The "Comparisons" column indicates the number of studies with direct comparisons between each intervention and control. Estimates for interventions with k=0 are derived entirely from indirect evidence within the network.

4.2.3 Network meta-analysis model only containing studies in which studies with small N were excluded. Studies with N size lower than interquartile range (N=45) defined as small.

The network was moderately well connected, only one subnetwork was present. 3/12 interventions only had one direct comparison (were not strongly attached). The model contained 140 studies, 12 treatments and 154 pairwise comparisons. The design-by-treatment interaction model suggested global inconsistency in the network tau^2 = 0.107; tau = 0.327; I^2 = 76.8% [72.6%; 80.2%]). However, under the assumption of a full design-by-treatment interaction random effects model, Q decreased considerably, and between-design inconsistency was no longer significant (Q = 10.42, p = 0.885).

ACT was no longer significantly more effective than control. The top ranked interventions were consistent with the main NMA, however yoga changed rank to first position, and EXPSY moved down to second, likely due to the large confidence interval.

SFigure 5

Sensitivity Analysis: Forest Plot of Network Meta-Analysis Excluding Small Sample Size Studies. Studies with N size lower than interquartile range (N=45) defined as small and excluded.

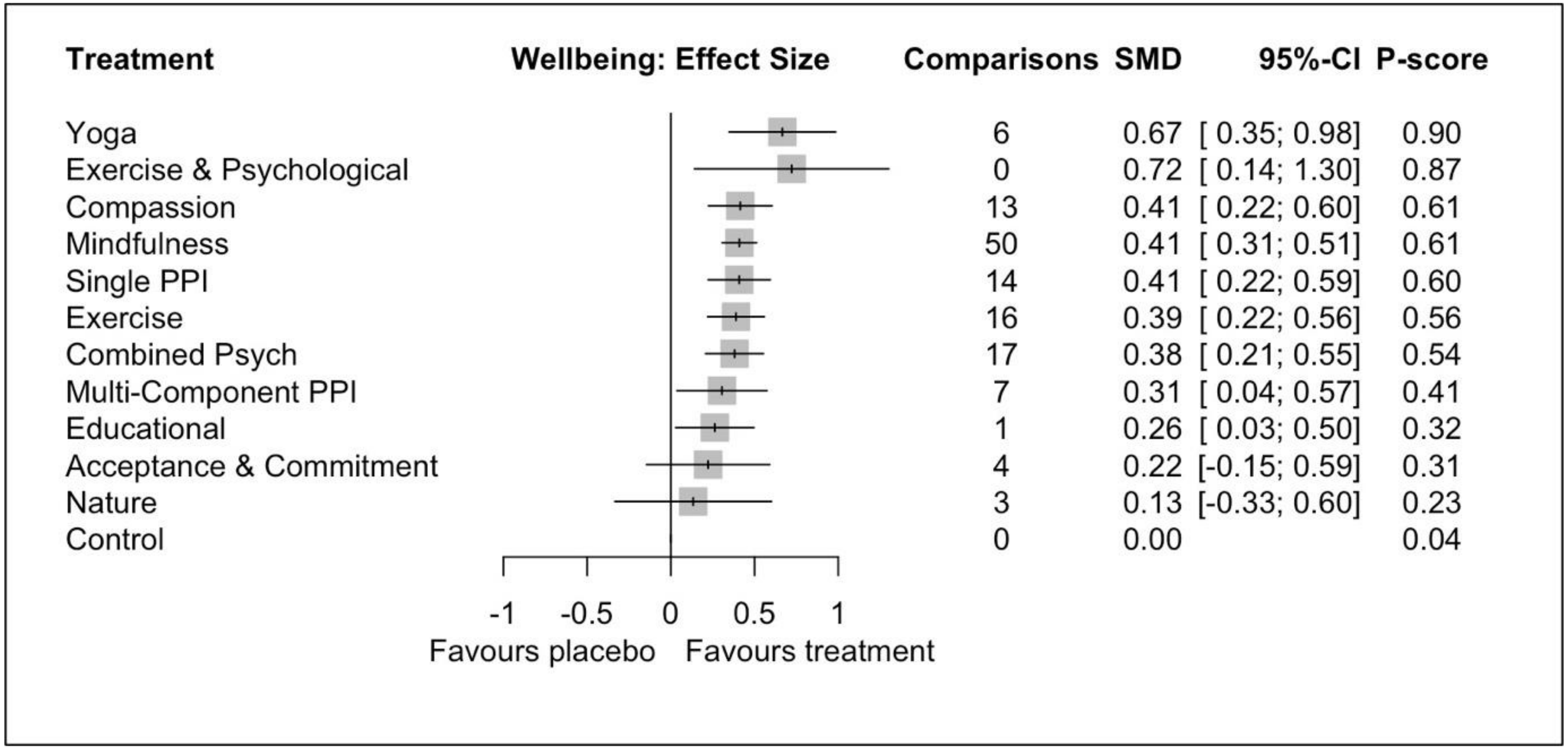

Note: Interventions ordered by P-score ranking. The "Comparisons" column indicates the number of studies with direct comparisons between each intervention and control. Estimates for interventions with k=0 are derived entirely from indirect evidence within the network.

4.2.4. Network meta-analysis model including grey literature

In response to reviewer feedback, additional searches were conducted in August 2025 to identify studies in the grey literature for inclusion. We searched clinicaltrials.gov, National Institute for Health and Care Research database (includes funded, but not always published trials), ProQuest dissertations and screened reference lists of relevant reviews.

Seven additional trials identified from grey literature searches were included (Bull-Beddows, 2020; Linford, 2020; Pitman, 2022; Prasek, 2015; Prudenzi, 2021; Salmon, 2004; Leininger, 2021). These studies added 709 participants across intervention and control groups. Specifically, they contributed additional comparisons for mindfulness (n = 213 participants), compassion (n = 103), exercise (n = 26), acceptance and commitment therapy (n = 52), and education interventions (n = 90).

The network remained well connected, with all 12 intervention nodes retained and 156 pairwise comparisons. The design-by-treatment interaction model suggested global inconsistency ($\tau^2 = 0.096$; $\tau = 0.310$; $I^2 = 75.9\%$ [72.1%; 79.4%]), but under the full design-by-treatment random effects model, Q decreased and between-design inconsistency was not significant ($Q = 11.28$, $p = 0.891$).

Across interventions, SMDs shifted only marginally (≤0.03) and 95% confidence intervals overlapped substantially with those in the main analysis. Treatment rankings were minimally affected: for example, compassion rose slightly above mindfulness, but both interventions remained within a similar band of effect size with overlapping CIs (SMD = 0.43–0.48).

Overall, the inclusion of grey literature did not change the pattern of results or substantive conclusions. These findings suggest that our primary NMA results are robust to the inclusion of unpublished or non-traditionally disseminated trials.

SFigure 6. Sensitivity Analysis: Forest Plot of Network Meta-Analysis Including Grey Literature Studies.

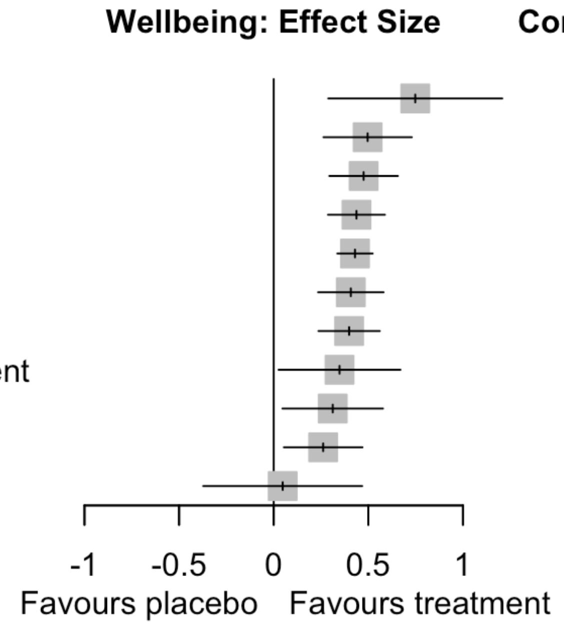

Treatment	Comparisons	SMD	95%-CI	P-score	Participants
Exercise & Psychological	1	0.75	[0.29; 1.21]	0.93	106
Yoga	12	0.50	[0.26; 0.73]	0.73	329
Compassion	16	0.48	[0.29; 0.66]	0.71	1122
Exercise	23	0.44	[0.29; 0.59]	0.63	1804
Mindfulness	68	0.43	[0.34; 0.52]	0.62	4823
Single PPI	18	0.41	[0.23; 0.58]	0.56	1435
Combined Psych	21	0.40	[0.24; 0.56]	0.54	1281
Acceptance & Commitment	6	0.35	[0.03; 0.67]	0.46	805
Multi-Component PPI	8	0.31	[0.05; 0.58]	0.38	441
Educational	1	0.26	[0.05; 0.47]	0.28	2250
Nature	4	0.05	[-0.37; 0.47]	0.12	123

Note: Interventions ordered by P-score ranking. The “Comparisons” column indicates the number of studies with direct comparisons between each intervention and control.

Reference List of Additional Grey Literature Included

Bull-Beddows, R. (2019). *Exploring the mechanisms in which a digital mindfulness-based intervention can help reduce stress and burnout among teachers* (Doctoral thesis, University of Southampton). University of Southampton Research Repository. https://eprints.soton.ac.uk/439321/1/Ryan_Bull_Beddows_Doctoral_Thesis_Manuscipt_18022020_e_Copy.pdf

Leininger, L. (2021). *A randomized controlled trial of a brief online mindfulness intervention for stress reduction among healthcare providers* [Clinical trial, unpublished]. ClinicalTrials.gov. https://clinicaltrials.gov/study/NCT03929393

Linford, L. B. (2020). *Efficacy of an online self-compassion training for improving well-being and body image: A randomized waitlist-controlled trial* (Master's thesis, Brigham Young University). ScholarsArchive. https://scholarsarchive.byu.edu/etd/9092

Pitman, N. J. (2022). *Mindfulness in the perinatal period* (Doctoral thesis, Canterbury Christ Church University). Canterbury Christ Church University Research Repository. https://repository.canterbury.ac.uk

Prasek, A. L. (2015). *Randomized controlled trial to evaluate a self-guided, web-based mindfulness program for stress reduction and wellbeing promotion* (Doctoral dissertation, University of Minnesota). University Digital Conservancy. https://conservancy.umn.edu/items/show/175228

Prudenzi, A. (2021). *Mental health and burnout in healthcare professionals: Exploring the role of psychological flexibility* (Doctoral dissertation, University of Leeds). White Rose eTheses Online. https://etheses.whiterose.ac.uk/29386

Salmon, P. G. (2004). *A comparison of brief, single sessions of physical activity and relaxation/meditation on affective responses of female undergraduates* (Master's thesis, University of Louisville). ThinkIR. https://doi.org/10.18297/etd/1254

4.3.5. Sensitivity Analysis: EXPSY vs Control

Only one trial directly compared combined exercise and psychological interventions (EXPSY) with control (Edwards & Loprinzi, 2019). This study (based on 110 university students completing a 10-minute walking plus mindfulness intervention) produced a non-significant direct effect estimate compared to control (SMD = –0.83, 95% CI: –1.72 to 0.07).

The pooled NMA estimate for EXPSY vs control was –0.73 (95% CI: –1.20 to –0.27), favoring EXPSY. Removing Edwards & Loprinzi (2019) in a sensitivity analysis produced a nearly identical result (–0.75, 95% CI: –1.34 to –0.15).

Thus, the EXPSY vs control estimate was not driven by the single direct comparison but was supported through indirect evidence in the broader network.

Additional evidence came indirectly from two studies that compared EXPSY with exercise alone: Sturm et al. (8-week “awe walks” in older adults) and Lee et al. (12-week walking plus positive education in retired adults). Both showed psychological benefits of the combined approach over exercise alone, but neither included comparisons with control or psychological interventions alone.

5 Confidence in network-meta analysis (CINEMA) judgements

Comparison	N of studies	Within-study bias	Reporting bias	Indirectness	Imprecision	Heterogeneity	Incoherence	Confidence rating
Acceptance & Commitment:Control	5	Some concerns	Low risk	Major concerns	No concerns	Major concerns	No concerns	Low
Combined Psych:Control	21	Some concerns	Low risk	Major concerns	No concerns	Major concerns	No concerns	Moderate
Combined Psych:Educational	3	Some concerns	Some concerns	Major concerns	Some concerns	Some concerns	No concerns	Moderate
Combined Psych:Exercise	1	Some concerns	Low risk	Major concerns	Some concerns	Some concerns	No concerns	Moderate
Combined Psych:Mindfulness	2	Major concerns	Low risk	Major concerns	Some concerns	Some concerns	No concerns	Moderate

Compassion:Control	15	Major concerns	High risk	Major concerns	No concerns	Major concerns	No concerns	Very low
Compassion:Exercise	1	Major concerns	Low risk	Major concerns	Major concerns	No concerns	No concerns	Low
Compassion:Mindfulness	1	Major concerns	Low risk	Major concerns	Major concerns	No concerns	No concerns	Low
Compassion:Multi-Component PPI	1	Major concerns	Low risk	Major concerns	Some concerns	Some concerns	No concerns	Moderate
Compassion:Single PPI	2	Major concerns	Low risk	Major concerns	Major concerns	No concerns	No concerns	Low
Control:Educational	1	Major concerns	Low risk	Major concerns	No concerns	Major concerns	No concerns	Low
Control:Exercise	23	Major concerns	Some concerns	Some concerns	No concerns	Major concerns	No concerns	Moderate
Control:Exercise & Psychological	1	Major concerns	Low risk	Major concerns	No concerns	Some concerns	No concerns	Moderate
Control:Mindfulness	65	Major concerns	Some concerns	Major concerns	No concerns	Major concerns	No concerns	Low
Control:Multi-Component PPI	8	Major concerns	Low risk	Major concerns	No concerns	Major concerns	No concerns	Low
Control:Nature	4	Some concerns	Low risk	Major concerns	Major concerns	No concerns	No concerns	Moderate
Control:Single PPI	18	Major concerns	High risk	Major concerns	No concerns	Major concerns	No concerns	Very low
Control:Yoga	12	Major concerns	Low risk	Some concerns	No concerns	Major concerns	No concerns	Moderate
Educational:Exercise	5	Major concerns	Low risk	Some concerns	Some concerns	Some concerns	No concerns	Moderate
Educational:Mindfulness	5	Major concerns	Low risk	Major concerns	Some concerns	Some concerns	No concerns	Moderate
Educational:Single PPI	1	Major concerns	Low risk	Major concerns	Some concerns	Some concerns	No concerns	Moderate
Exercise:Exercise & Psychological	3	Major concerns	Low risk	No concerns	Some concerns	Some concerns	No concerns	Moderate
Exercise:Mindfulness	1	Major concerns	Low risk	Major concerns	Some concerns	Some concerns	No concerns	Moderate
Exercise:Yoga	3	Major concerns	Low risk	Some concerns	Some concerns	Some concerns	No concerns	Moderate

Exercise & Psychological:Mindfulness	1	Major concerns	Low risk	Major concerns	Some concerns	Some concerns	No concerns	Moderate
Mindfulness:Single PPI	2	Major concerns	Low risk	Major concerns	Some concerns	Some concerns	No concerns	Moderate
Mindfulness:Yoga	2	Major concerns	Low risk	Some concerns	Some concerns	Some concerns	No concerns	Moderate
Multi-Component PPI:Single PPI	1	Major concerns	Low risk	Major concerns	Major concerns	No concerns	No concerns	Low
Acceptance & Commitment:Combined Psych	0	Some concerns	Low risk	Major concerns	Major concerns	No concerns	No concerns	Moderate
Acceptance & Commitment:Compassion	0	Major concerns	Low risk	Major concerns	Major concerns	No concerns	No concerns	Moderate
Acceptance & Commitment:Educational	0	Some concerns	Low risk	Major concerns	Major concerns	No concerns	No concerns	Moderate
Acceptance & Commitment:Exercise	0	Some concerns	Low risk	Major concerns	Major concerns	No concerns	No concerns	Moderate
Acceptance & Commitment:Mindfulness	0	Major concerns	Low risk	Major concerns	Major concerns	No concerns	No concerns	Moderate
Acceptance & Commitment:Multi-Component PPI	0	Major concerns	Low risk	Major concerns	Major concerns	No concerns	No concerns	Moderate
Acceptance & Commitment:Nature	0	Some concerns	Low risk	Major concerns	Major concerns	No concerns	No concerns	Moderate
Acceptance & Commitment:Single PPI	0	Major concerns	Low risk	Major concerns	Major concerns	No concerns	No concerns	Moderate
Acceptance & Commitment:Yoga	0	Major concerns	Low risk	Major concerns	Major concerns	No concerns	No concerns	Moderate
Combined Psych:Compassion	0	Major concerns	Low risk	Major concerns	Some concerns	Some concerns	No concerns	Moderate
Combined Psych:Exercise & Psychological	0	Major concerns	Low risk	Major concerns	Some concerns	Some concerns	No concerns	Moderate
Combined Psych:Multi-Component PPI	0	Major concerns	Low risk	Major concerns	Major concerns	No concerns	No concerns	Low
Combined Psych:Nature	0	Some concerns	Low risk	Major concerns	Some concerns	Some concerns	No concerns	Moderate
Combined Psych:Single PPI	0	Major concerns	Low risk	Major concerns	Major concerns	No concerns	No concerns	Low

Combined Psych:Yoga	0	Major concerns	Low risk	Some concerns	Some concerns	Some concerns	No concerns	Moderate
Compassion:Educational	0	Major concerns	Low risk	Major concerns	Some concerns	Some concerns	No concerns	Moderate
Compassion:Exercise & Psychological	0	Major concerns	Low risk	Major concerns	Major concerns	No concerns	No concerns	Moderate
Compassion:Nature	0	Major concerns	Low risk	Major concerns	Some concerns	Some concerns	No concerns	Moderate
Compassion:Yoga	0	Major concerns	Low risk	Some concerns	Major concerns	No concerns	No concerns	Low
Educational:Exercise & Psychological	0	Major concerns	Low risk	Major concerns	Some concerns	Some concerns	No concerns	Moderate
Educational:Multi-Component PPI	0	Major concerns	Low risk	Major concerns	Major concerns	No concerns	No concerns	Low
Educational:Nature	0	Major concerns	Low risk	Major concerns	Major concerns	No concerns	No concerns	Low
Educational:Yoga	0	Major concerns	Low risk	Some concerns	Some concerns	Some concerns	No concerns	Moderate
Exercise:Multi-Component PPI	0	Major concerns	Low risk	Major concerns	Major concerns	No concerns	No concerns	Low
Exercise:Nature	0	Major concerns	Low risk	Major concerns	Some concerns	Some concerns	No concerns	Moderate
Exercise:Single PPI	0	Major concerns	Low risk	Major concerns	Major concerns	No concerns	No concerns	Low
Exercise & Psychological:Multi-Component PPI	0	Major concerns	Low risk	Major concerns	Some concerns	Some concerns	No concerns	Moderate
Exercise & Psychological:Nature	0	Major concerns	Low risk	Major concerns	No concerns	Major concerns	No concerns	Low
Exercise & Psychological:Single PPI	0	Major concerns	Low risk	Major concerns	Some concerns	Some concerns	No concerns	Moderate
Exercise & Psychological:Yoga	0	Major concerns	Low risk	Some concerns	Major concerns	No concerns	No concerns	Moderate
Mindfulness:Multi-Component PPI	0	Major concerns	Low risk	Major concerns	Some concerns	Some concerns	No concerns	Moderate
Mindfulness:Nature	0	Major concerns	Low risk	Major concerns	Some concerns	Some concerns	No concerns	Moderate
Multi-Component PPI:Nature	0	Major concerns	Low risk	Major concerns	Major concerns	No concerns	No concerns	Low

Multi-Component PPI:Yoga	0	Major concerns	Low risk	Major concerns	Some concerns	Some concerns	No concerns	Moderate
Nature:Single PPI	0	Major concerns	Low risk	Major concerns	Some concerns	Some concerns	No concerns	Moderate
Nature:Yoga	0	Major concerns	Low risk	Major concerns	Some concerns	Some concerns	No concerns	Moderate
Single PPI:Yoga	0	Major concerns	Low risk	Some concerns	Major concerns	No concerns	No concerns	Moderate

6. Publication Bias

SFigure 7
Funnel plot of potential publication bias for all direct comparisons

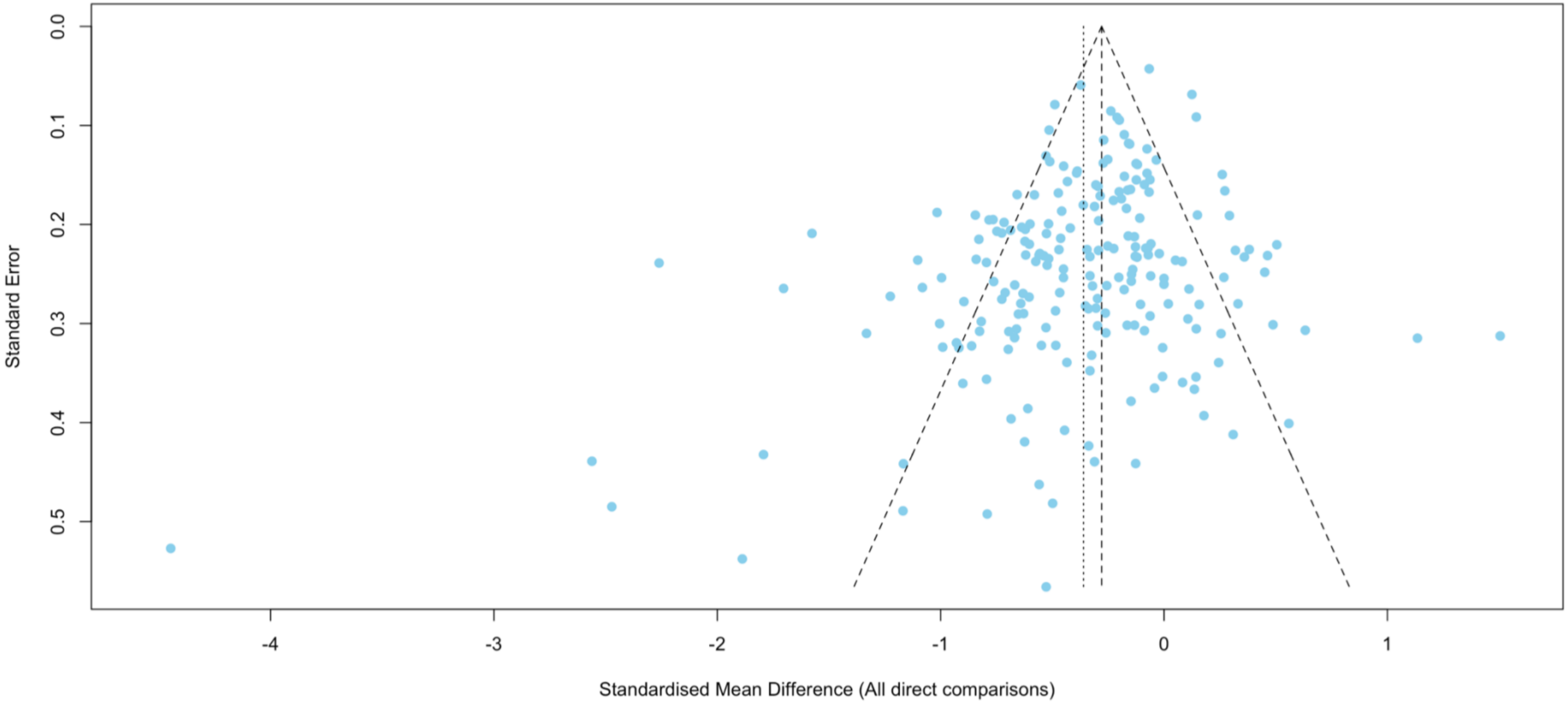

Note: Each point represents an individual study. The dashed lines indicate pseudo 95% confidence limits around the pooled effect estimate. Egger's regression test confirmed funnel plot asymmetry ($t(206) = –4.16$, $p < .001$, bias estimate = –1.20, SE = 0.29).

SFigure 8

Funnel plot of potential publication bias for all direct comparisons with grey literature included

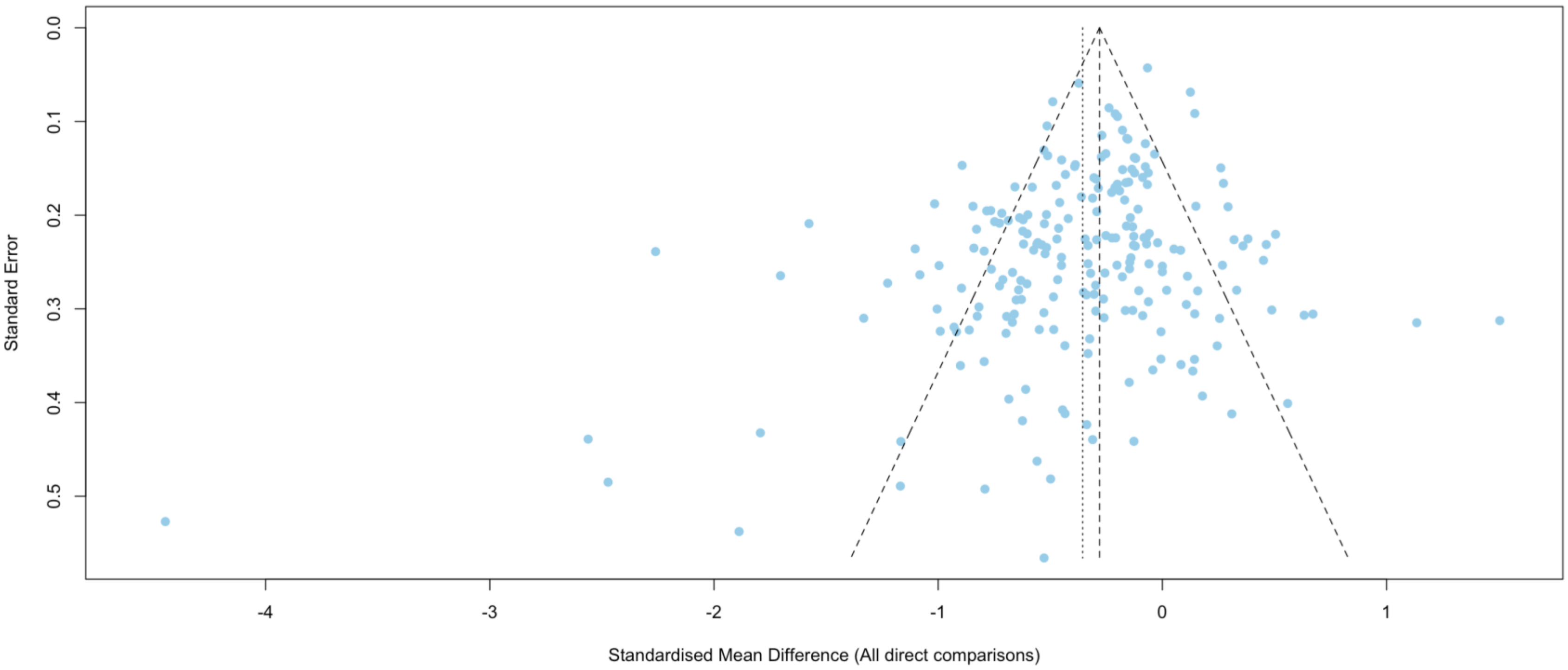

Note: Each point represents an individual study. The dashed lines indicate pseudo 95% confidence limits around the pooled effect estimate. Egger's regression test remained significant for asymmetry (t(213) = –3.98, p < .001, bias estimate = –1.14, SE = 0.29).
