# Supplementary material for: A systematic review and network meta-analysis of randomized controlled trials of well-being-focused interventions
Source: Nat Hum Behav. 2026 Jan 2;10(4):715–26. doi: 10.1038/s41562-025-02369-1 (PMC13120999; doi:10.1038/s41562-025-02369-1)
Supplement: Supplementary file 2 — Reporting Summary [file 41562_2025_2369_MOESM2_ESM.pdf]

nature portfolio

Corresponding author(s): Lowri Wilkie and Andrew Kemp

Last updated by author(s): 10/7/2025

Reporting Summary

Nature Portfolio wishes to improve the reproducibility of the work that we publish. This form provides structure for consistency and transparency in reporting. For further information on Nature Portfolio policies, see our Editorial Policies and the Editorial Policy Checklist.

Statistics

For all statistical analyses, confirm that the following items are present in the figure legend, table legend, main text, or Methods section.

n/a	Confirmed	
☒	☐	The exact sample size (*n*) for each experimental group/condition, given as a discrete number and unit of measurement
☒	☐	A statement on whether measurements were taken from distinct samples or whether the same sample was measured repeatedly
☐	☒	The statistical test(s) used AND whether they are one- or two-sided<br>*Only common tests should be described solely by name; describe more complex techniques in the Methods section.*
☐	☒	A description of all covariates tested
☒	☐	A description of any assumptions or corrections, such as tests of normality and adjustment for multiple comparisons
☐	☒	A full description of the statistical parameters including central tendency (e.g. means) or other basic estimates (e.g. regression coefficient) AND variation (e.g. standard deviation) or associated estimates of uncertainty (e.g. confidence intervals)
☐	☒	For null hypothesis testing, the test statistic (e.g. *F*, *t*, *r*) with confidence intervals, effect sizes, degrees of freedom and *P* value noted<br>*Give P values as exact values whenever suitable.*
☒	☐	For Bayesian analysis, information on the choice of priors and Markov chain Monte Carlo settings
☐	☒	For hierarchical and complex designs, identification of the appropriate level for tests and full reporting of outcomes
☐	☒	Estimates of effect sizes (e.g. Cohen's *d*, Pearson's *r*), indicating how they were calculated

Our web collection on statistics for biologists contains articles on many of the points above.

Software and code

Policy information about availability of computer code

Data collection	Data collection, study screening, and extraction were conducted using Covidence (https://www.covidence.org/). No custom software was developed for data collection.
Data analysis	All analyses were conducted in R (version 4.4.2) using the packages netmeta, meta, metafor, dplyr, and ggplot2. Network meta-analyses were implemented using the netmeta package, and confidence in estimates was assessed using CINeMA (https://cinema.ispm.unibe.ch/). All code are available in the project's Open Science Framework (OSF) repository [https://osf.io/nz59j/?view_only=30f14278418f454e8c6ee297493f2c39].

For manuscripts utilizing custom algorithms or software that are central to the research but not yet described in published literature, software must be made available to editors and reviewers. We strongly encourage code deposition in a community repository (e.g. GitHub). See the Nature Portfolio guidelines for submitting code & software for further information.

Data

Policy information about availability of data

All manuscripts must include a data availability statement. This statement should provide the following information, where applicable:

- Accession codes, unique identifiers, or web links for publicly available datasets
- A description of any restrictions on data availability
- For clinical datasets or third party data, please ensure that the statement adheres to our policy

All data supporting the findings of this study are available in the Open Science Framework (OSF) repository at: https://osf.io/nz59j/?view_only=30f14278418f454e8c6ee297493f2c39. The repository includes the dataset extracted from all included trials used for network meta-analysis and meta-regression.

Research involving human participants, their data, or biological material

Policy information about studies with human participants or human data. See also policy information about sex, gender (identity/presentation), and sexual orientation and race, ethnicity and racism.

Reporting on sex and gender	This study is a network meta-analysis that synthesises data from previously published randomised controlled trials (RCTs). No new data were collected from human participants, and therefore, ethical oversight, recruitment, and participant-level demographic reporting do not apply.
Reporting on race, ethnicity, or other socially relevant groupings	*Please specify the socially constructed or socially relevant categorization variable(s) used in your manuscript and explain why they were used. Please note that such variables should not be used as proxies for other socially constructed/relevant variables (for example, race or ethnicity should not be used as a proxy for socioeconomic status).* *Provide clear definitions of the relevant terms used, how they were provided (by the participants/respondents, the researchers, or third parties), and the method(s) used to classify people into the different categories (e.g. self-report, census or administrative data, social media data, etc.)* *Please provide details about how you controlled for confounding variables in your analyses.*
Population characteristics	See Behavioural & social sciences study design
Recruitment	No human participants were recruited
Ethics oversight	No ethical approval was required. Only published data was used.

Note that full information on the approval of the study protocol must also be provided in the manuscript.

Field-specific reporting

Please select the one below that is the best fit for your research. If you are not sure, read the appropriate sections before making your selection.

☐ Life sciences ☒ Behavioural & social sciences ☐ Ecological, evolutionary & environmental sciences

For a reference copy of the document with all sections, see nature.com/documents/nr-reporting-summary-flat.pdf

Behavioural & social sciences study design

All studies must disclose on these points even when the disclosure is negative.

Study description	This study is a network meta-analysis (NMA) synthesizing quantitative data from previously published randomised controlled trials (RCTs). No new data were collected, and therefore, information regarding sampling, data collection, timing, exclusions, non-participation, and randomisation refers to the original studies included.
Research sample	The mean age across studies was 38.3 years (range 18–82). Studies were primarily conducted in Western countries (79%), most frequently the USA, China, the UK, Australia, and Spain. Approximately half of studies reported participant sex, with most indicating a majority of women. Fewer than 10% of studies reported participant ethnicity, and reporting categories were inconsistent. All included trials recruited non-clinical adult samples.
Sampling strategy	No new participant sampling was conducted. Sample sizes were determined by the original trials and varied across studies. The final analysis included 183 RCTs comprising 22,811 adult participants. The large aggregated sample provides sufficient statistical power for estimating comparative effects.
Data collection	This study did not involve the collection of new participant data. All data were extracted from previously published randomised controlled trials identified through systematic database searches. Screening and data extraction were conducted independently by two reviewers using Covidence, with discrepancies resolved through discussion. Extracted data included study characteristics, intervention details, sample size, and wellbeing outcome measures. As all analyses were based on published summary statistics, no

participants or researchers were present during data collection, and blinding was not applicable.

Timing	The primary literature search was conducted in March 2023 across multiple electronic databases (PsycINFO, PubMed/MEDLINE, Scopus, and Web of Science) using predefined search terms for wellbeing interventions in adult populations. In response to reviewer feedback, an updated search was conducted in August 2025 to identify unpublished or grey literature. This supplementary search included ClinicalTrials.gov, the National Institute for Health and Care Research (NIHR) database, ProQuest Dissertations, and reference lists of relevant reviews. Both searches followed PRISMA 2020 guidelines and used identical inclusion and exclusion criteria.
Data exclusions	Of 9,105 unique records screened, 183 randomised controlled trials met the inclusion criteria and were included in the final network meta-analysis (see Table 3 for eligibility criteria and Figure 1 for the PRISMA flow diagram). Following data extraction, 66 studies were excluded during standard network refinement to maintain model assumptions and connectivity (see Supplementary Information 2.2.3).
Non-participation	Not applicable. This study is a systematic review and network meta-analysis of previously published trials. No participants were directly recruited or followed by the authors.
Randomization	All included studies were randomised controlled trials (RCTs) in which participants were allocated to experimental and control conditions using randomisation procedures as reported in the original studies. No new participant allocation occurred in this network meta-analysis.

Reporting for specific materials, systems and methods

We require information from authors about some types of materials, experimental systems and methods used in many studies. Here, indicate whether each material, system or method listed is relevant to your study. If you are not sure if a list item applies to your research, read the appropriate section before selecting a response.

Materials & experimental systems

n/a	Involved in the study
☒	☐ Antibodies
☒	☐ Eukaryotic cell lines
☒	☐ Palaeontology and archaeology
☒	☐ Animals and other organisms
☒	☐ Clinical data
☒	☐ Dual use research of concern
☒	☐ Plants

Methods

n/a	Involved in the study
☒	☐ ChIP-seq
☒	☐ Flow cytometry
☒	☐ MRI-based neuroimaging

Plants

Seed stocks	*Report on the source of all seed stocks or other plant material used. If applicable, state the seed stock centre and catalogue number. If plant specimens were collected from the field, describe the collection location, date and sampling procedures.*
Novel plant genotypes	*Describe the methods by which all novel plant genotypes were produced. This includes those generated by transgenic approaches, gene editing, chemical/radiation-based mutagenesis and hybridization. For transgenic lines, describe the transformation method, the number of independent lines analyzed and the generation upon which experiments were performed. For gene-edited lines, describe the editor used, the endogenous sequence targeted for editing, the targeting guide RNA sequence (if applicable) and how the editor was applied.*
Authentication	*Describe any authentication procedures for each seed stock used or novel genotype generated. Describe any experiments used to assess the effect of a mutation and, where applicable, how potential secondary effects (e.g. second site T-DNA insertions, mosiacism, off-target gene editing) were examined.*
